# GPX8 regulates clear cell renal cell carcinoma tumorigenesis through promoting lipogenesis by NNMT

**DOI:** 10.1186/s13046-023-02607-2

**Published:** 2023-02-07

**Authors:** Tin Tin Manh Nguyen, Thi Ha Nguyen, Han Sun Kim, Thien T. P. Dao, Yechan Moon, Munjun Seo, Sunmi Kang, Van-Hieu Mai, Yong Jin An, Cho-Rok Jung, Jin-Mo Kim, Sunghyouk Park

**Affiliations:** 1 Natural Products Research Institute, College of Pharmacy, Seoul National University, Seoul, 08826 Republic of Korea; 2 Molecular Biology Department, School of Medicine, Vietnam National University, Ho Chi Minh City, 70000 Vietnam; 3 Korea Research Institute of Bioscience and Biotechnology (KRIBB), Daejeon, 34141 Republic of Korea; 4 Department of Functional Genomics, Korea University of Science and Technology (UST), Daejeon, 34113 Republic of Korea

**Keywords:** Clear cell renal cell carcinoma (ccRCC), GPX8, NNMT, AMPK, De novo lipogenesis (DNL)

## Abstract

**Background:**

Clear cell renal cell carcinoma (ccRCC), with its hallmark phenotype of high cytosolic lipid content, is considered a metabolic cancer. Despite the implication of this lipid-rich phenotype in ccRCC tumorigenesis, the roles and regulators of de novo lipid synthesis (DNL) in ccRCC remain largely unexplained.

**Methods:**

Our bioinformatic screening focused on ccRCC-lipid phenotypes identified glutathione peroxidase 8 (GPX8), as a clinically relevant upstream regulator of DNL. GPX8 genetic silencing was performed with CRISPR-Cas9 or shRNA in ccRCC cell lines to dissect its roles. Untargeted metabolomics, RNA-seq analyses, and other biochemical assays (e.g., lipid droplets staining, fatty acid uptake, cell proliferation, xenograft, etc.) were carried out to investigate the GPX8’s involvement in lipid metabolism and tumorigenesis in ccRCC. The lipid metabolic function of GPX8 and its downstream were also measured by isotope-tracing-based DNL flux measurement.

**Results:**

GPX8 knockout or downregulation substantially reduced lipid droplet levels (independent of lipid uptake), fatty acid de novo synthesis, triglyceride esterification in vitro, and tumor growth in vivo. The downstream regulator was identified as nicotinamide N-methyltransferase (NNMT): its knockdown phenocopied, and its expression rescued, GPX8 silencing both in vitro and in vivo. Mechanically, GPX8 regulated NNMT via IL6-STAT3 signaling, and blocking this axis suppressed ccRCC survival by activating AMPK. Notably, neither the GPX8-NNMT axis nor the DNL flux was affected by the von Hippel Lindau (VHL) status, the conventional regulator of ccRCC high lipid content.

**Conclusions:**

Taken together, our findings unravel the roles of the VHL-independent GPX8-NNMT axis in ccRCC lipid metabolism as related to the phenotypes and growth of ccRCC, which may be targeted for therapeutic purposes.

**Graphical abstract:**

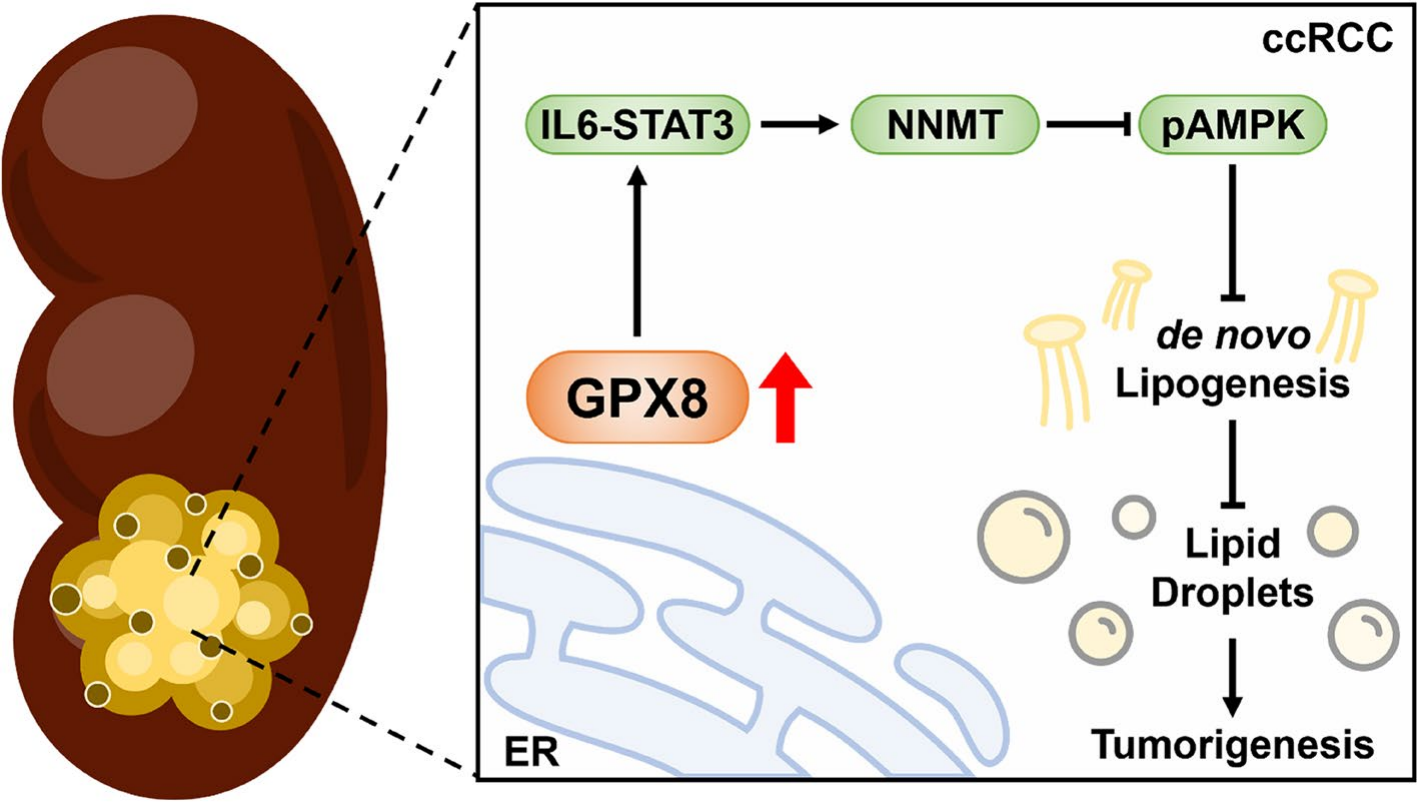

**Supplementary Information:**

The online version contains supplementary material available at 10.1186/s13046-023-02607-2.

## Background

ccRCC is the most common and aggressive type of kidney cancer (KC), accounting for about 70% cases and the majority of deaths for KC [1]. Among the various factors contributing to ccRCC, suppression of VHL, either by a genetic or epigenetic mechanism, is the most prevalent, its inactivation being observed in 75% of ccRCC cases [2]. VHL loss stabilizes hypoxia-induced factors (HIFs), which leads to activation of an array of tumor-growth-supporting pathways such as aerobic glycolysis, vasculogenesis, and cell survival [3]. Although the VHL-HIF axis is a hallmark of ccRCC, VHL inactivation alone does not seem to be sufficient for ccRCC tumorigenesis, as it takes decades for people with germline VHL mutations to develop ccRCC [4], and knockout of VHL in mouse kidney has failed to induce ccRCC histology [5].

ccRCC is sometimes called a metabolic disease, since it entails substantial metabolic alterations including enhanced glycolysis and TCA-cycle suppression [6]. In line with this, a prominent feature of ccRCC is its “clear cell” microscopic character arising from the high content of glycogen and lipids in the cytosol. Although the roles of glycogen in ccRCC are disputable [7, 8], the high lipid level has been implicated in the tumor progression and pathophysiology of ccRCC [9–11]. The levels of enzymes such as FASN or SCD1 in lipid synthetic pathways correlate with tumor growth and poor patient survival/outcome [12, 13]. Genetic or pharmacological suppression of SCD1 and DGAT, enzymes that increase the unsaturated fatty acid ratio in lipid droplets, inhibits tumor growth [14]. Emerging work also has suggested that FAO reduction by CPT1A downregulation [11] or lipid-deposition process enhancement by PLIN2 [9], both mediated by mutated VHL and HIFs, contributes to the high lipid content in ccRCC. However, the roles or regulators of DNL, another important aspect of the characteristic high-lipid feature, in relation to ccRCC phenotypes and aggressiveness remain poorly understood.

GPX8 belongs to the glutathione peroxidase family along with GPX1-4, 6, and GPX5, 7. The former group has a selenocysteine at the active site and catalyzes reactive oxygen species (ROS) detoxification at the expense of reduced glutathione, whereas the latter group and GPX8 with a cysteine may carry out additional functions [15]. GPX7 and GPX8 are endoplasmic reticulum (ER)-resident proteins with high sequence similarity, except that GPX8 has an additional membrane-tethering sequence and serine instead of otherwise-conserved glutamine at the catalytic tetrad. Due to the high sequence similarity, they have common functions in which they remove peroxides formed during oxidative protein folding in the ER by protein disulfide isomerase and ERO1A [16]. Nevertheless, the phenotypes of the knockout mice of each gene are different: GPX7 KO mice feature abnormal immune regulation and systemic increases in ROS level [17], whereas GPX8-KO mice exhibit no apparent phenotypes under conditions lacking immune stress [18]. GPX8 has been the least-characterized GPX, though several reports have implicated it in diverse cellular processes such as calcium homeostasis in ER [19], microsomal lipid composition regulation [20], and protection against chemical-induced colitis [18]. Interestingly, recent studies are beginning to shed light on GPX8’s involvement in cancer by showing its roles in the activation of wingless/integrated (WNT) signaling [21] or regulation of invasion and migration [22]. However, if and how GPX8 can affect cancer growth through regulation of tissue-specific lipid phenotypes has not been demonstrated.

By performing bioinformatic screening tuned to ccRCC-specific phenotypes of high glycogen and lipid contents, we identified GPX8 as a clinically relevant regulator of ccRCC phenotype and growth. Mechanistic work has delineated the functional effectors of GPX8, such as NNMT and AMPK. As blocking of the GPX8-NNMT axis inhibits growth and lipogenesis in ccRCC cells, GPX8-NNMT axis may represent a new phenotype-directed therapeutic target for ccRCC.

## Methods

### Cell culture and isotope labeling experiment condition

Human ccRCC cell lines 786O (from ATCC), Caki1, and A498 (Korean Cell Line Bank) were cultured in DMEM (786O and Caki1) or MEM (A498), supplemented with 10% FBS, 1% penicillin–streptomycin. All cells were routinely tested for Mycoplasma contamination (MycoAlert; tested every three months). For the DNL experiment, cells were plated at 50–70% confluency in 100 mm dishes overnight. On the next day, the medium was changed to 10 mM U^13^C-glucose (Cambridge Isotope Laboratories, USA) supplemented with 10% dialyzed FBS (Gibco), and cells were cultured for 9 h before extraction. To prepare the lipid-depleted FBS for the lipid-depleted experiment, lipids in FBS were removed by stripping with silica gel following the previous reference [23].

### Plasmids, CRISPR-Cas9, lentiviral production, and transduction

For genetic knockout, CRISPR-Cas9 vector pSpU6Cas9-2A-Puro (from Macrogen) was cloned with human sgRNAs targeting GPX8 (AGCTGCAAGAGGCTCCATGTTGG). Plasmids were transfected using lipofectamine 3000 (Thermo Fisher Scientific, US) under the manufacturer’s instruction. Transfected cells were selected by puromycin and were plated by serial dilution to obtain the single-cell colonies which were subsequently validated by western blot analysis. For overexpression experiments, pCMV6-Entry Mammalian Expression Vector (control, CAT#: PS100001) and pCMV6-Entry-Myc-DDK-tag carrying NNMT (CAT#: RC200641) and VHL (CAT#: RC216151) were obtained from Origene (US). Cells were transfected by indicated plasmids using lipofectamine 3000 and then selected by neomycin to generate stable cell lines. The VHL overexpressing-cells were selected with neomycin and confirmed to express VHL stably over extended passages. The lentiviral vectors expressing shNNMT#1 and #2 (HSH011860-LVRU6H) were obtained from Genecopoeia (US) including shRNA control vector. pLKO-Tet-On (tetracycline-inducible shRNA) was cloned with the shRNA targeting GPX8 (#1 CCGGCCATGAGGGTTTGGTCTCATTCTCGAGAATGAGACCAAACCCTCATGGTTTTTT; #2 CCGGGCCATTGCGTTTCTAATAGAACTCGAGTTCTATTAGAAACGCAATGGCTTTTTT). Lentivirus was produced by transfecting HEK293T with lentiviral shRNA vector, PLP-1, PLP-2, and VSV-G using lipofectamine 3000. Lentiviral particles were harvested at 24 h and 48 h post transfections. For viral transduction, cells were incubated with lentivirus in the presence of 10 μg/mL polybrene for 48 h followed by 24 h recovery in fresh medium before antibiotic selection (hygromycin for shNNMT and puromycin for shGPX8). We used different gene manipulation approaches in downregulating GPX8 for Caki1 and 786O cells to avoid any possible off-target effects associated with either CRISPR-Cas9 or shRNA method. Similar results with these different approaches would help solidify the conclusion by excluding off-target effects of each method.

### Cell proliferation, wound-healing assay, and colony assays

Cell proliferation and cytotoxicity assay were performed using Cell Counting Kit-8 (CCK8 reagent, Dojindo, Kumamoto, Japan). Cells were plated in 96-well plates at 2000 or 3000 cells per well for cell proliferation or cytotoxicity assay, respectively, and allowed to attach overnight. The media were changed according to the condition of the experiment on the next day. Following the manufacturer’s instruction, cells at indicated time points were treated with 10 µL of CCK8 and incubated for 1 h before spectrophotometric quantitation at 450 nm. The absorbance values of each cell type on different days were divided by the average value from the control cells on day 2 to yield the relative cell growth. Experiments were performed in triplicates. For wound healing assay, cells were seeded to reach full confluence in 12-well plates which were then scratched. The media was changed to that supplemented with 2% FBS. The migration into the gap was imaged over 24 h and 48 h using inverted microscopy. The in vitro tumorigenesis was evaluated by the clonogenic assay. Cells (for Caki1 WT, GPX8 KO, or GPX8 KO/NNMT OE) were plated in 6-well plates at 200 cells per well and cultured in DMEM supplemented with 10% FBS for about 2 weeks. Then, cells were stained with 0.5% crystal violet in 20% methanol for 10 min. The number of colonies was quantified using the NIH Image J program.

### Survival analysis

TCGA gene expression data used for survival analysis was identical to the one used in the bioinformatics screening. TCGA survival information was obtained from TCGA-CDR [24]. The survival analysis was done with “survival” and “survminer” package in R. The stratification of patients was made by setting optimal cutpoint with a minimum proportion of 0.3 per group, using the surv_cutpoint function.

### RNA-seq and functional analysis

RNA sequencing (RNA-Seq) analysis was performed by Illumina NovaSeq 6000 system (by Macrogen Company). Briefly, samples (triplicates) were prepared with the TruSeq Stranded mRNA LT Sample Prep Kit (Illumina) from RNA, and sequencing was performed using the NovaSeq 6000 S4 Reagent Kit (Illumina) with paired-end reads, all according to the manufacturer’s recommendations. Data quality was checked using FastQC v0.11.7 (http://www.bioinformatics.babraham.ac.uk/projects/fastqc/). Further adapter trimming was performed using Trimmomatic 0.38. Low quality ends with < Q30 were trimmed and reads shorter than 36 bases after adapter trimming were discarded. Raw sequencing data were mapped to the human reference genome (GRCh37.73) with HISAT2 (ver. 2.1.0) (https://ccb.jhu.edu/software/hisat2/index.shtml). The transcripts were assembled by StringTie (ver. 2.1.3b) from the read alignment data. The raw counts of genes were normalized by DESeq2 package (R ver. 4.1.1) and further analyzed to generate the differentially expressed genes (DEGs). Functional analysis was performed using ClusterProfiler package (R ver. 4.1.1) on DEGs with |Log2(fold change)|≥ 2 and *p*-value ≤ 0.05. Volcano plots were generated using GraphPad Prism ver. 9.3.0. GSEA was analyzed using an unfiltered gene list in the GSEA (ver. 4.0.3) with GO (Gene Ontology) and PID (Pathway Interaction Database).

### Bioinformatics screening and analysis

CCLE gene expression (raw count) and metabolomics data were downloaded from CCLE website (http://portals.broadinstitue.org/ccle/data, downloaded on May 2019). The raw counts were normalized using DESeq2 package in R. For total triacylglycerol, we used mean values of all triacylglycerol species. For bioinformatic screening, we downloaded the data from TCGA PanCanAtlas (https://gdc.cancer.gov/about-data/publications/pancanatlas). According to the cBioportal where the TCGA PanCancer data is also stored, RSEM unit was used. When we checked the minimum value of the downloaded data, it was -0.9912106, as opposed to the expected value of 0. As adding 1 to values close to this minimal value before log2-normalization could introduce a big bias, we added 2 before performing log2 normalization. For other clinical data analysis, we obtained data from xena.ucsc.edu and cbioportal.org in FPKM and RSEM units, respectively, and added 1 before log2 transformation. For the cutoffs used for gene screening, we used 0.0001 for FDR and 0.15 for the absolute value of rho. The Venn diagrams were drawn using the Vennerable package in R. All analysis was done using R 4.0.3.

### Single-cell RNA-seq analysis

ScRNA-seq data from Young and colleagues [25] were reanalyzed following the Seurat pipeline for quality control, clustering, normalization, batch correction, and visualization provided in the same study and in the Seurat guideline (http://satijalab.org/seurat/) [26]. We extracted the single cell data from WT-VHL ccRCC (BulkID PD36793a) and MT-VHL ccRCC patients (BulkID PD37228c) for comparison (one patient for each group). The supplement data from Young and colleagues explicitly describes the sample information of the dataset. For quality control, cells with less than 200 RNA features and a high mitochondrial fraction (> 5%) were removed. The data were normalized by dividing by the total unique molecular identifiers (UMI) counts of each cell and then natural-log transformed by using “NormalizeData” function with the Seurat scale factor (default value, 10,000). Next, the batch correction was performed following the standard workflow of Seurat package in which the method first identifies pairs of cells across the dataset that are in the matched biological state (to find integration anchors) and then corrects the technical differences between datasets. Cells were clustered and visualized in two dimensions through the Uniform Manifold Approximation and Projection (UMAP) plots. The tumor cluster was annotated by differential markers (CA9, NDUFA4L2) according to the original paper.

### Extraction method for NMR/MS analysis

For extraction, cells were extracted by (1) washing 3 times with chilled PBS, (2) quenching in 80% MeOH, (3) scraping and transferring into new EP tubes, (4) extracting using freeze–thaw cycle (3 times) in DW:MeOH:CHCl_3_ (1:2:2). The mixture was then centrifuged at 14,000 g at 4 °C for 20 min. The separated upper and lower layers were dried under a vacuum evaporator and stored at –80 °C until further analysis. For protein quantification, the dry pellets were used for a standard BCA analysis using PierceTM BCA protein assay kit (Thermo Fisher Scientific).

### NMR experiments

The hydrophobic extracts from cells in DNL experiments were dissolved in CDCl_3_ for the NMR experiment. The 2D spectra were obtained using J-scaled distortion-free 2D-^1^H-^13^C-HSQC [27] in 800 MHz Bruker Avance (Bruker Biospin GmbH, Rheinstetten, Germany) equipped with a cryogenic triple resonance probe at the College of Pharmacy, Seoul National University (Seoul, Korea). NMR acquisition was acquired at 25 °C according to the following parameters: Spectral width (SW), 40 ppm along the ^13^C and 16 ppm along the ^1^H dimensions; O1P, 4.7 ppm; O2P, 27 ppm; Time domain (TD), 2048 (for proton) × 300 (for carbon) increments with scaling factor, 6; Number of scan (NS), 4; Acquisition time, 23 min for each spectrum; Interscan delay, 1.0 s. NMR data were processed and analyzed using the Topspin 3.6.2 software provided by Bruker. For quantification, the peak intensity was used and normalized by total protein content.

### LC–MS-based targeted and untargeted metabolomics

The dried hydrophilic and hydrophobic extracts were reconstituted in a mixture of DW:ACN (50 μL, 1:1 v/v) or IPA:ACN (50 μL, 1:1 v/v) respectively and centrifuged at 14,000 g at 4 °C for 5 min.

For LC–MS condition used in untargeted metabolomics experiment, the separation was performed using Zic-pHilic column (150 mm × 2.1 mm, particle size: 5 μm, Merck) at 40 °C by Acquity UPLC Waters coupled with Q Exactive™ Focus Hybrid Quadrupole-Orbitrap™ Mass Spectrometer (Thermo Fisher Scientific). The mobile phases were 10 mM (NH_4_)_2_CO_3_ (A) and ACN (B) with the gradient as follows: 20% A from 0 to 2 min, then increase gradually to 80% for 17 min and keep it for 4 min. Set 20% A at 23.1 min followed by 2 min equilibrium with a 0.15 mL/min flow rate. The Q Exactive Focus MS system was equipped with Heated electrospray ionization (HESI-II) probe with the following settings: Sheath gas, 40 mL/min; Auxiliary gas, 10 mL/min, heated to 250 °C; Sweep gas, 2 mL/min; Spray voltage, 2.5 kV, followed by the capillary temperature at 256 °C; S-lens RF level, 50; Both negative and positive modes were employed. The LC–MS data were corrected by an internal standard (^13^C_5_-^15^N_2_-glutamine) and normalized by protein content.

For palmitate isotope-tracing experiment, BEH C18 was used to separate palmitate with buffer A (10 mM NH_4_AC in ACN:DW (6:4)) and buffer B (10 mM NH_4_AC in IPA:ACN (9:1)) with the following gradient: 85% A with for 1 min, then gradually decrease to 18% in 16.2 and maintain it for 2 min before setting A at 1% for 0.8 min. The equilibrium was set for 4 min from 21.1 to 25 min. The flow rate and column temperature were set at 0.4 mL/min and 35 °C, respectively. We used Thermo-Vanquish LC connected with MS—Q Exactive Plus with resolution 280 K to resolve the ^2^H and ^13^C fatty acid mass isotopomers as the following setting: Sheath gas, 50 mL/min; Auxiliary gas = 12.5 mL/min, heated to 425 °C; Sweep gas, 2 mL/min; Spray voltage, 2.5 kV, followed by the capillary temperature at 262.5 °C; S-lens RF level, 55; Negative mode.

Targeted metabolomics was analyzed by 4000 QTRAP or Agilent 6460 Triple Quad mass spectrometer system coupled with Agilent 1290 HPLC system. BEH amide column (100 × 2.1 mm, 1.7 µm; Waters) was eluted with buffer A (10 mM NH_4_AC in 100% DW + 10 mM NH_4_OH) and B (10 mM NH_4_AC in 80% ACN: 20% DW + 10 mM NH_4_OH) as following gradient: Set 0% A with the flowrate 0.2 mL/min from 0 to 2 min, then increase gradually to 40% for 5.5 min and keep for 3.5 min. Decrease to 0% A and increase the flow rate to 0.3 mL/min at 11.5 min followed by 4.5 min equilibrium and then return to 0.2 mL/min flow rate.

For the isotope tracing experiment, data were acquired, and isotopologue peaks were extracted using Xcalibur ver. 2.8 (Thermo Fisher Scientific) or El-maven ver. 0.12.0.

### Metabolomics analysis

For untargeted metabolomics, raw data were processed using MzMine (ver. 2.53). In brief, the following modules were used: Mass detection (with noise level, 1e4), ADAP chromatogram builder, Wavelets (ADAP) for deconvolution, Isotopic peak grouper, RANSAC alignment, Gap filling (same RT and m/z range), all with 5 ppm for mass tolerance and 0.25 min for RT tolerance. The final peak list included features found in at least 50% of the samples. Metabolites with a *p*-value ≤ 0.05 and a |log2(fold change)|≥ 0.5 were considered to be significantly changed, which were further annotated using MS/MS spectra databases of HMDB (The Human Metabolome Database) and analyzed using Metaboanalyst 4.0 (http://www.metaboanalyst.ca/).

### mRNA quantification

Total RNA was extracted using Easy-spin RNA extraction kit (Intron, Sungnam, Korea). cDNA was synthesized from 2 μg RNA using the High-Capacity cDNA Reverse Transcription Kit (Lot No. 1304185, Applied Biosystems, Inc., USA). qPCR reactions were performed in triplicate in an Applied Biosystems 7300 PCR machine with SYBR green-based detection (iTaq TM Universal SYBR Green Supermix, Cat. No. 172–5120, Bio-Rad, USA). The ΔCt values were calculated by normalization with β-actin. β-actin primer was obtained from Bioneer (Daejon, Korea).

### BODIPY assay for lipid droplet

For lipid droplet staining, we adapted a previous protocol [28]. Cells were plated on the culture slide for 2 more days after reaching confluence. Cells were then washed with DPBS and incubated in 2 μM BODIPY 493/503 (cat. D3922, Life Technology) in serum-free medium for 15 min at 37 °C before fixing with 4% paraformaldehyde followed by staining with DAPI for nuclei. For measuring the lipid uptake, cells were incubated with 6 μM BODIPY 500/510 C1, C12 (cat. D3822) in serum-free medium for 30 min at 37 °C. Fixed cells were then visualized by Leica confocal microscope. All samples from the same experiment were imaged by using the same settings (gain, laser power). We quantified lipid staining via counting the number of lipid droplets by using NIH Image J program in at least 20 cells and then calculated the average number of lipid droplets per cell in each condition. For lipid uptake, fluorescence intensity was measured as follows. Cells were suspended and incubated with 6 μM BODIPY 500/510 C1, C12 for 30 min at 37 °C. Fixed cells were then analyzed using Flow Cytometer (BD, FACS Calibur) and data were analyzed using FlowJo software. All experiments were done at least in triplicates.

### Western blot analysis

Cells were scraped and homogenized in RIPA buffer added with protease and phosphatase inhibitors. Lysates were centrifuged at 14,000 g for 20 min at 4 °C and the protein content was normalized by BCA assay. Samples were processed with SDS buffer in 95 °C for 10 min, resolved in SDS-PAGE gel, and then transferred onto nitrocellulose or PVDF membranes using the wet tank transfer system (Bio-rad). Blots were incubated with primary antibodies (diluted 1:1,000 in 1% bovine serum albumin (BSA)) for overnight at 4 °C. For detection, horseradish peroxidase-conjugated anti rabbit or anti mouse secondary antibody were used, followed by exposure to chemiluminescent reagent (Abfrontier WESTSAVE ECL Solution, Cat: F-QC0106). Blots were then imaged using a digital imaging machine (LAS 4000, GE Healthcare, or Chemidoc XRS).

### Immunohistochemistry experiment

Xenograft tumor tissue slides or kidney carcinoma tissue microarray slide containing 48 samples (10 cases of clear cell carcinoma, 10 granular cell carcinoma, and 4 normal kidney tissue; duplicate) in 1.5-mm diameter / five-micron core (KD482, US Biomax, Inc., Derwood, MD, USA) embedded in paraffin was examined for the GPX8 expression. Tissue sections were immunolabeled for GPX8 (MyBioSource, San Diego, CA, USA) using heat-induced antigen retrieval and standard immunohistochemical procedures and 3,3’-diaminobenzidine as chromagen. Tissue sections were probed overnight with primary antibodies diluted in phosphate-buffered saline (PBS) containing 0.5% bovine serum albumin (BSA) and were probed with primary antibodies (4 °C, overnight). The samples were incubated overnight with HRP enzyme conjugated rabbit secondary antibodies (Abcam, Cambridge, MA, USA) and were developed using DAB (3,3′-diaminobenzidine) substrate kit (Abcam, Cambridge, MA, USA). All immunohistochemically labeled slides were lightly counter-stained with hematoxylin.

### Oil red O staining

The frozen sections of fresh xenograft tumor tissues were stained with Oil Red O. Briefly, cryopreserved tissues embedded with optical cutting temperature compound were cut into 15-μm sections and fixed in 10% formalin for 20 min. Sections were washed in 60% isopropanol for 5 min, then incubated in 0.5% Oil Red O (Sigma, USA) in 100% isopropanol working solution for 30 min. The stained tissues were washed with water before counterstaining in hematoxylin for 1 min. The slides were mounted with aqueous mounting media and examined under light microscopy.

### Animal experiment

Male BALB/c nude mice (age, 6 weeks; weight, 20–25 g) were purchased from Orient Bio Laboratory Animal Research Center Co., Ltd (Seoul, Korea) and allowed to acclimatize (*n* = 5 per cage) for a week. All mice were kept in laminar-flow cabinets under specific pathogen-free conditions. For xenograft models of human ccRCC cell line, Caki1 at the cell density of 5 × 10^6^ cells mixed with Matrigel (BD Biosciences, San Jose, CA) in 200 μL PBS with 1:1 ratio was subcutaneously implanted onto BALB/c nude mice. Following the procedure, mice were monitored to ensure the procedure was well tolerated. The length (L) and width (W) of each tumor was measured by vernier caliper and the tumor volume was calculated by the formula V = (L × WxW)/2.

### Statistical analysis

Each biological experiment was conducted at least in three replicates (*n ≥ 3*), unless otherwise stated, and the results of the replicates were analyzed with an appropriate method (unpaired Student’s *t*-test or two-way ANOVA) to establish statistical significance. The GraphPad Prism 9.3.0 software was used to perform all statistical analyses. Error bars represent the standard deviation (SD) from the mean and *P*-values were calculated as indicated in figure legends.

## Results

### GPX8 is associated with higher grade and poor prognosis in ccRCC

**Fig. 1:**
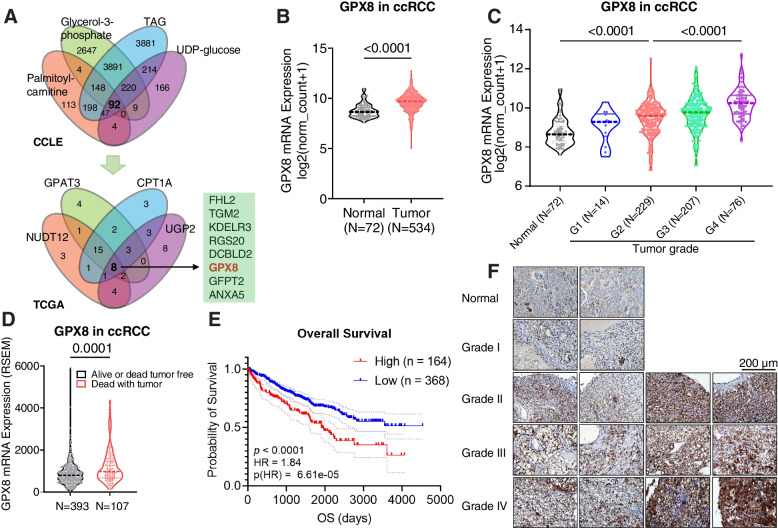
GPX8 is associated with higher grade and poor prognosis in ccRCC **A**, Two-tiered bioinformatic screening to find genes correlated with metabolites (top; CCLE database) and genes (bottom; TCGA-KIRC (Kidney renal clear cell carcinoma) database) of glycogen and lipid metabolism in ccRCC. The metabolites and genes used as queries are indicated in each Venn diagram. The criteria were set to FDR < 0.0001 and |r|< 0.15. The numbers in the Venn diagram represent the numbers of genes that meet the criteria. B-D, Violin plots for the mRNA expression levels of GPX8 according to (**B**) normal vs. tumor, (**C**) neoplasm histologic grade, and (**D**) alive or dead-tumor-free group vs. dead-with-tumor group among ccRCC patients. The mRNA expression values were obtained from the TCGA-KIRC dataset. *P*-values were determined by Mann–Whitney U test. **E**, Overall survival according to GPX8 mRNA expression for ccRCC patients from TCGA-KIRC database. **F**, GPX8 IHC staining for tumor array sections (KD482_biomax) from ccRCC patients. Magnification 400X

To find a gene associated with the metabolic characteristics of ccRCC, we performed two-tiered bioinformatic screening (Figs. 1A and S1A). First, we employed a metabolite-driven approach using the Cancer Cell Line Encyclopedia (CCLE) database with key metabolites of the two characteristic phenotypes of ccRCC. Correlation analysis with UDP-glucose for glycogen metabolism or palmitoyl-carnitine, total triacylglycerol (TAG), and glycerol-3-phosphate for lipid metabolism yielded a total of 92 significantly correlated genes. Second, from the gene-driven filtering, 8 out of the 92 were found to be correlated with key enzymes in glycogen (UGP2 and NUDT12) and lipid (CPT1A and GPAT3) metabolisms. Among the 8 genes, FHL2 and TGM2 already have been studied for ccRCC [29, 30], and, among the rest, GPX8 exhibited the highest cancer versus normal expression ratio with a high-enough expression level (Figs. 1B and S1B). Therefore, we evaluated the clinical relevance of GPX8 in ccRCC using clinical parameters in the TCGA database and our own immunohistochemical results. First, GPX8 mRNA expression proportionally increased according to the grades of kidney tumors (Fig. 1C), which was confirmed at the protein level with an independent large proteomic database (CPTAC) (Fig. S1C). Second, as for prognosis, the GPX8 expression was higher in patients who had died from tumors than those who survived them or died without them (Fig. 1D). Third, patients with new tumor events after primary therapies had higher GPX8 levels than those without them (Fig. S1D). Fourth, patients with a higher GPX8 level exhibited poor prognosis in terms of overall survival, progression-free interval, disease-free interval, and disease-specific survival (Figs. 1E and S1E-G). Finally, our own immunohistochemical (IHC) staining of ccRCC tissues also confirmed the GPX8-tumor grade correlation and higher expression of GPX8 in cancer than in normal tissues (Fig. 1F). Taken together, GPX8, identified from the ccRCC-phenotype-driven bioinformatic screening, exhibited strong relevance to ccRCC clinical outcomes.

### *GPX8 is involved in ccRCC growth* in vitro and in vivo

**Fig. 2:**
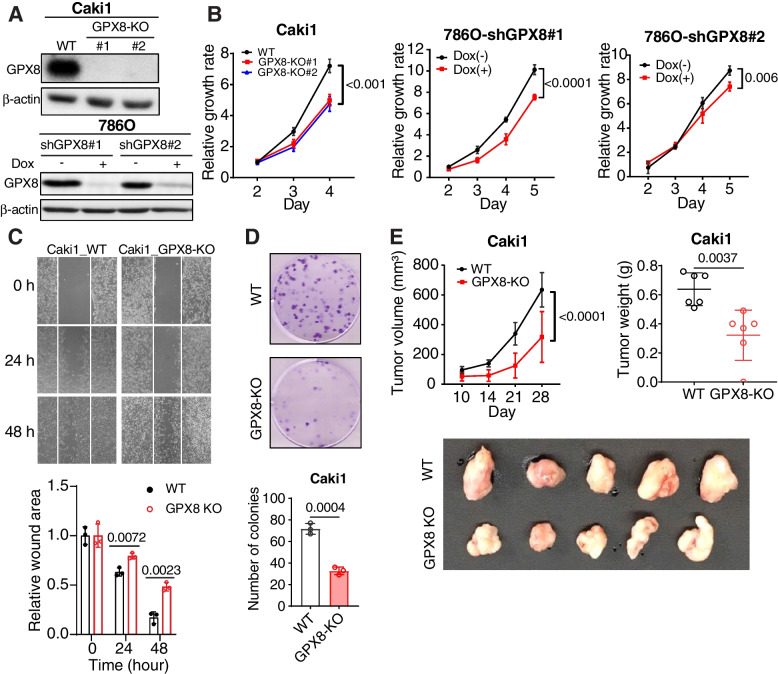
GPX8 is involved in ccRCC cell growth in vitro and in vivo A-D, The effect of GPX8 KO in Caki1 and shGPX8 in 786O cells in vitro. Western blot analysis of GPX8 protein expression (A), relative growth rates measured by CCK8 kit (B), migrating cells in scratch assay at 0, 24, and 48 h (top) and bar graphs for relative wound areas for WT and GPX8-KO Caki1 cells (bottom) (*n* = 3) (C), and clonogenic assay after plating 200 cells in 6-well plate for 2 weeks (top) and bar graphs for number of colonies of WT and GPX8-KO Caki1 (bottom) (*n* = 3) (D). E, Tumor growths by WT and GPX8-KO Caki1 cells xenografted in nude mice (*n* = 6). One mouse in the GPX8-KO group did not develop a visible tumor, and the tumor photo shows 5 tumors. See Fig. S2C for the photos for the whole body mouse images. Tumor volume (top, left), tumor weight (top, right), and photograph of tumors (bottom) obtained at day 28 after implantation. Data presented in panels (B), (C), (D), and (E) are means ± SD (*n* ≥ 3). *P*-value was calculated by two-way ANOVA with Geisser–Greenhouse correction for (B) and (E) (top, left panel); unpaired *t*-test for (C), (D) and (E) (top, right panel)

To study the causal involvement of GPX8, we silenced its expression in Caki1 (using CRISPR-Cas9) and 786O (using dox-inducible shRNA) ccRCC cell lines that carry wild type (WT) and mutant (MT) VHL, respectively (Fig. 2A). These cell lines exhibited significantly reduced growth upon GPX8 knockout (GPX8-KO) or knockdown (shGPX8) compared with control cells (Fig. 2B). In addition, GPX8-KO Caki1 cells showed reduced migratory activity in a scratch assay as well as fewer and smaller colony formations in a clonogenic assay (Figs. 2C and D) relative to the WT cells, suggesting a lower tumorigenicity. These results were consistent in shGPX8 786O cells (Figs. S2A and B). In vivo xenograft experimentation also showed smaller tumor volumes and lower tumor weights from GPX8-KO than from WT (Figs. 2E and S2C). These data confirm that GPX8 is involved in ccRCC tumorigenesis.

### GPX8 regulates lipid metabolism in ccRCC

**Fig. 3:**
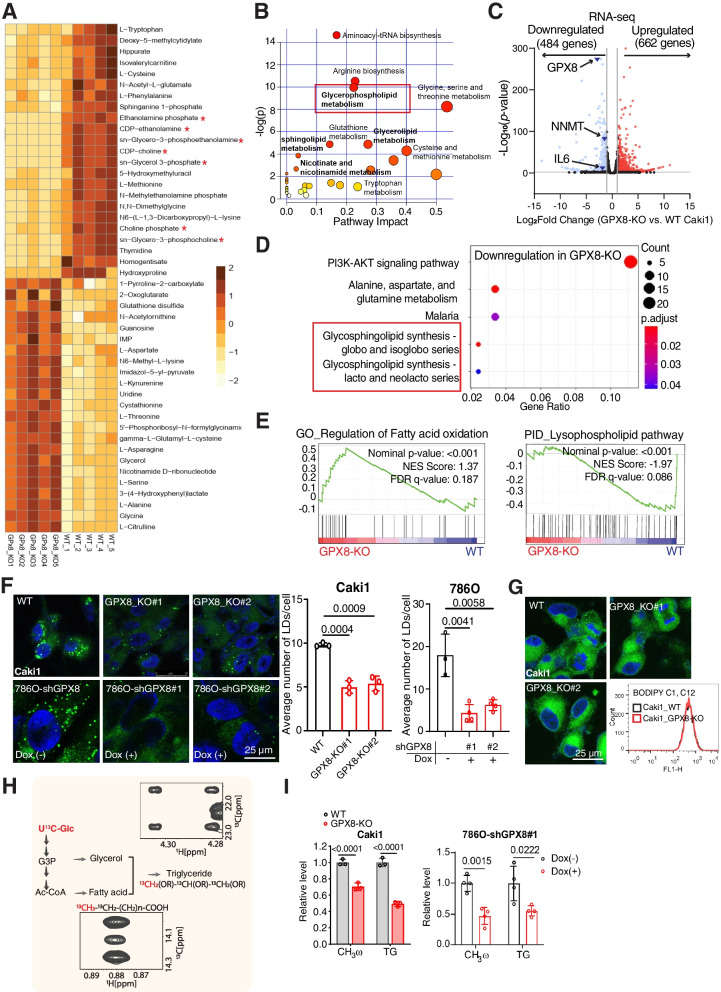
GPX8 regulates lipid metabolism in ccRCC A-B, Heat map (A) and metabolic pathway enrichment analysis (B) of significantly different metabolite levels from untargeted metabolomics comparing GPX8-KO vs. WT Caki1 (*n* = 5). Red asterisks indicate metabolites related to the glycerophospholipid pathway (A). C-D, Volcano plot (C) and pathway analysis of downregulated genes (KEGG pathway) (D) from RNA-seq data for GPX8-KO vs. WT Caki1. Lipid metabolism-related pathways are in red box (D). Criteria: |Log2(fold change)|≥ 2 with *P*-value ≤ 0.05. E, GSEA analysis of GPX8 correlation with lipid metabolism pathways: regulation of FAO (from Gene ontology) and lysophospholipid pathway (from Pathway Interaction Database). F, Representative pictures (left) of neutral lipid BODIPY 493/503 staining from WT vs. GPX8-KO Caki1 and shGPX8 786O cells with or without doxycycline (100 ng/mL). Quantitation of the lipid droplet (right) (*n* = 3): The number of lipid droplets per cell was quantified as detailed in the Methods section. G, Representative pictures of BODIPY 500/510 C1, C12 staining for WT and GPX8-KO Caki1 cells for lipid uptake as measured by fluorescent intensity with flow cytometry (right). H-I, FA de novo synthesis (CH3ω) and triacylglycerol synthesis (esterified-glycerol) from U^13^C-glucose with NMR (H), and bar graphs for their relative levels normalized by total protein level (BCA) comparing WT vs. GPX8-KO Caki1 cells (left) and shGPX8 786O cells with or without doxycycline (100 ng/mL) for 3 days (right) (I). Data presented in panels (F) and (I) are means ± SD (*n* ≥ 3). *P*-value was calculated by unpaired *t*-test

Next, the involvement of GPX8 in lipid metabolism, a characteristic phenotype of ccRCC, was investigated. A metabolomic comparison between WT and GPX8-KO cells showed a clear distinction (Fig. S3A) as well as a substantial decrease in intermediates for glycerophospholipid metabolism in the GPX8-KO cells (Figs. 3A and B). Subsequent RNA-seq transcriptomic results showed profound changes in gene expression, in which GPX8 KO cells exhibited 484 downregulated (including NNMT and IL-6, see below) and 662 upregulated genes compared to WT cells (Fig. 3C). Then, functional analysis indicated downregulation of glycosphingolipid biosynthesis (differentially expressed gene analysis; Fig. 3D) along with upregulation of FAO and downregulation of lysophospholipid pathways (gene set enrichment analysis; Fig. 3E). Lipid metabolism was further evaluated with additional, specific biochemical assays. Lipid droplets observed by confocal fluorescence were less numerous in GPX8-KO Caki1 as well as in shGPX8 786O cells (Fig. 3F). This effect seems due to endogenous cellular lipid metabolism rather than uptake of lipid species, as evidenced by the similar levels of cellular fluorescence upon the addition of a fatty acid-conjugated fluorescence probe to the media (Figs. 3G and S3B). Consistently, the lipid droplets almost disappeared in the GPX8-KO cell line grown in the lipid-depleted medium, whereas WT cells still maintained the lipid droplets at a slightly lower number (Fig. S3C), thus corroborating the roles of GPX8 in the DNL. Supporting this, DNL activity from U^13^C-glucose, as measured through CH_3_ω peaks in NMR [31], decreased by about 40% and 50% in the GPX8-KO Caki1 and shGPX8 786O cells, respectively (Figs. 3H and I). In addition, triacylglycerol formation through the esterification of fatty acid and glycerol decreased by about 50% in the GPX8-KO Caki1 and shGPX8 786O cells, as directly measured by the ^13^C-esterified-glycerol peaks in NMR (Figs. 3H and I). Pharmacologically, both C75 (FASN inhibitor) and TOFA (ACC inhibitor) significantly reduced the lipid droplet levels in the Caki1 and 786O ccRCC cell lines (Fig. S3D). As both inhibitors inhibited ccRCC cell survival concentration-dependently (Fig. S3E), DNL seems to be necessary for both lipid accumulation and survival of ccRCC. Overall, our multi-omics, tracer experiments, and biochemical results confirm the critical roles of GPX8 in ccRCC lipid accumulation through enhanced DNL.

### GPX8 enhances lipid accumulation by inhibiting AMPK

**Fig. 4:**
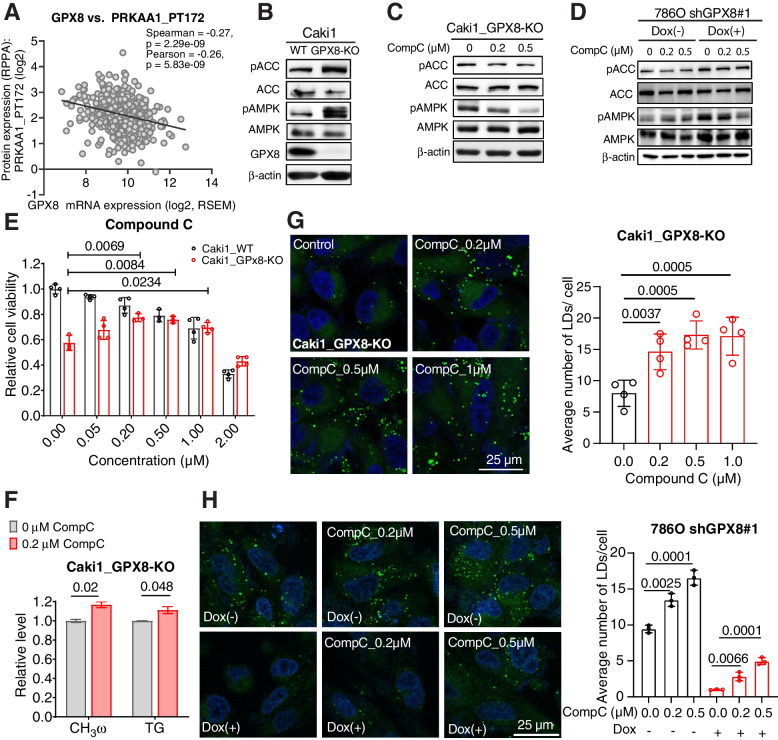
GPX8 enhances lipid accumulation by inhibiting AMPK. A, Correlation between GPX8 mRNA expression and phosphorylated AMPKα1 (PRKAA1_PT172) level obtained from Reverse Phase Protein Arrays (RPPA) data of TCGA-KIRC dataset. B-C, Western blot analysis for phosphorylated and total forms of ACC (Ser 79) and AMPK α1 (T183) α2 (T172) in GPX8 WT and KO cells (B) and effects of compound C on GPX8-KO Caki1 cells for 2 days (C). D, shGPX8 786O cells were incubated with or without doxycycline (100 ng/mL) for 3 days. These cells then were treated with compound C in a range of concentration for 2 days. Western blot analysis for the effects of compound C on phosphorylated forms of ACC (Ser 79) and AMPK α1 (T183) α2 (T172). E, Relative cell viability of WT and GPX8-KO Caki1 cells upon treatment of different concentrations of compound C for 3 days. F, FA de novo synthesis and triacylglycerol synthesis in GPX8-KO with and without compound C (0.2 µM) as in Fig. 3H. G, Representative pictures from quadruplicates (left) of neutral lipid BODIPY 493/503 staining of GPX8-KO Caki1 cells treated with different concentrations of compound C for 3 days. Quantitation of the lipid droplet (right) (*n* = 4) as in Fig. 3F. H, Representative pictures from triplicates (left) and quantitation (right) for lipid staining for shGPX8 786O cells as in Fig. 3F (*n* = 3). Data presented in panels (E), (F), (G), and (H) are means ± SD (*n* ≥ 3). *P*-values were determined by unpaired *t*-test

We then considered how GPX8 may regulate de novo lipogenic processes in ccRCC. We became particularly interested in AMPK, as it is a central player in metabolism and is negatively involved in progression/tumorigenesis of ccRCC [32]. Both AMPK expression and its phosphorylated form were correlated negatively with GPX8 but were positively associated with the overall survival of ccRCC patients (Figs. 4A, S4A and B). Experimentally, the knockout of GPX8 in Caki1 activated AMPK, as revealed by the increase in phosphorylated AMPK (pAMPK) and phosphorylated ACC (pACC) (Fig. 4B), which was also shown in the shGPX8 786O cells (Fig. S4C). This negative regulation of AMPK by GPX8 is consistent with the decreased DNL in GPX8-KO cells, as AMPK inhibits ACC, an important enzyme in DNL. Pharmacologically, the inhibition of AMPK with compound C, a widely used AMPK inhibitor [33], reversed the increase in pACC and pAMPK in the GPX8-KO Caki1 cells (Fig. 4C) and shGPX8 786O cells (Fig. 4D). Remarkably, it also rescued the growth inhibition by GPX8-KO concentration-dependently, despite its inherent toxicity to both WT and KO cells at higher concentrations (Fig. 4E). These results were consistent in shGPX8 786O cells (Fig. S4D). At the metabolic level, compound C reversed the metabolic changes by suppressing AMPK activation in GPX8-KO cells, where it increased both fatty acid and TG synthesis (Fig. 4F). These actions ultimately resulted in restored lipid accumulation in the GPX8-KO Caki1 and shGPX8 786O cells, without exerting much effect on the WT cells (Figs. 4G, H and S4E). Pharmacological effects were consistently observed in the knockdown of AMPK (siAMPK) in GPX8-KO and WT Caki1 cells (Figs. S4F and G). Finally, AICAR, a well-known activator of AMPK, substantially inhibited the growth of Caki1 cells (Fig. S4H). Collectively, both pharmacological and transcriptional inhibition of AMPK rescued growth and metabolic phenotypes of GPX8 loss, indicating that GPX8 exerts its effects in ccRCC through inhibition of AMPK, which is implicated in ccRCC growth and prognosis.

### NNMT mediates GPX8’s inhibition of AMPK

**Fig. 5:**
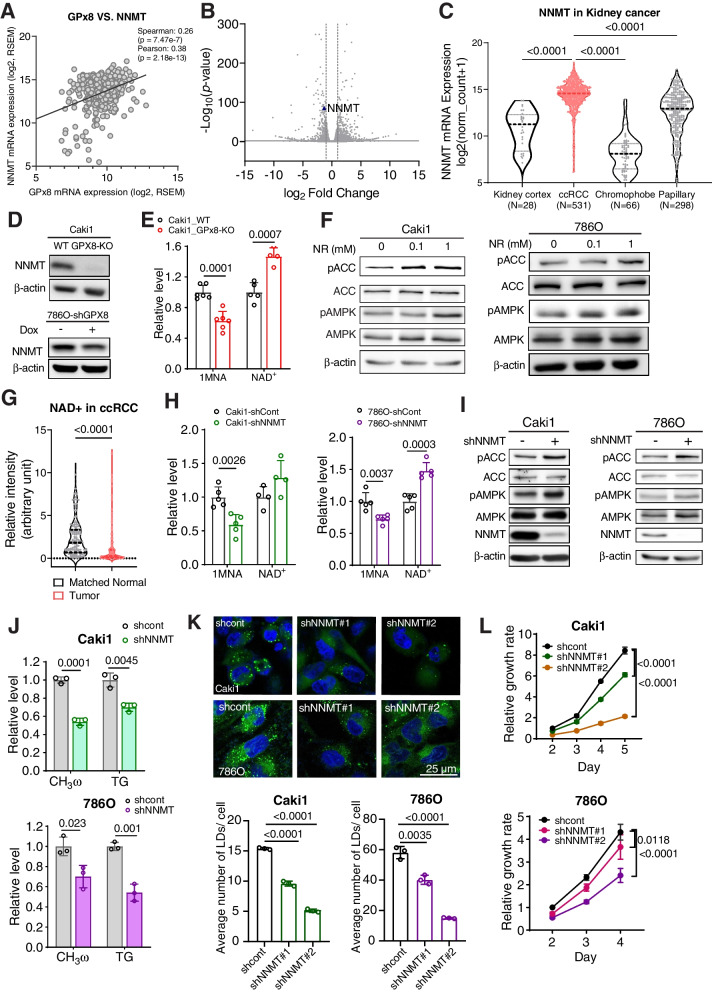
NNMT mediates GPX8’s inhibition of AMPK. A, Correlation between GPX8 and NNMT mRNA expression in ccRCC patients from TCGA-KIRC dataset. B, Volcano plot for NNMT mRNA expression from RNA-seq data comparing GPX8-KO vs. WT Caki1. C, NNMT expression in different subtypes of renal cancer and normal tissues from TCGA-KIRC. *P*-values were determined by Mann–Whitney U test. D, Western blot analysis of NNMT in GPX8-KO Caki1 and shGPX8 786O vs. control cells. E, Relative levels of 1MNA and NAD ^+^ from GPX8-KO and WT cells as measured by LC–MS/MS. F, Western blot analysis of total and phosphorylated forms of ACC (Ser 79) and AMPK α1 (T183) α2 (T172) in Caki1 and 786O upon NR treatment for 2 days. G, Relative levels of NAD ^+^ between tumor and matched normal tissues in ccRCC patients from previous dataset [35]. *P*-value was determined by Wilcoxon matched–paired signed-rank test. H–L, The effect of shNNMT in Caki1 and 786O cells. Relative levels of 1MNA and NAD ^+^ as measured by LC–MS/MS (H), western blot analysis of total and phosphorylated forms of ACC (Ser 79) and AMPK α1 (T183) α2 (T172) (I), FA de novo synthesis (CH3ω) and triacylglycerol synthesis (TG) (J), representative pictures (top) of neutral lipid BODIPY 493/503 staining from Caki1 shNNMT and 786O shNNMT with quantitation of the lipid droplet (bottom) (*n* = 3) as in Fig. 3F (K), and relative growth rates (L). *P*-value was calculated by two-way ANOVA with Geisser–Greenhouse correction (L). Western blot analysis was normalized by β-actin. Data from (E), (H), and (J) are normalized by total protein level. Data from (E), (H), (J), and (K) are means ± SD (*n* ≥ 3). *P*-values were determined by unpaired *t*-test

In trying to understand how GPX8 inhibits AMPK, we noticed “nicotinate and nicotinamide metabolism” in our metabolomic results (See Fig. 3B). In addition, a CCLE metabolomic database query for GPX8 returned 1-methylnicotinamide (1MNA) as the top hit with another unusually high and significant correlation (Spearman: r = 0.616, *p* = 1.517e-96) (Fig. S5A). These two metabolomic analyses led us to investigate NNMT that synthesizes 1MNA at the expense of NAD^+^. In a transcriptomic database (TCGA-KIRC), NNMT expression exhibited a significant positive correlation with GPX8 (Spearman: r = 0.260, *p* = 7.47e-7) (Fig. 5A) and significant negative correlations with pAMPKα1 (T172) (Spearman: r = -0.22, *p* = 1.166e-4) (Fig. S5B). Interestingly, a recent report suggested that NNMT inhibits AMPK in a non-cancer disease in liver [34], where NNMT expression is higher in normal than cancer tissues. In comparison, NNMT expression and its ratio to that in normal tissues in ccRCC patients were among the highest across many cancers (Fig. S5C). In our RNAseq data, NNMT was also lower in GPX8-KO cells (Fig. 5B). Phenotypically in ccRCC, NNMT expression was much higher in ccRCC than in normal tissues, its level being correlated with higher grades (Fig. S5D). In addition, NNMT expression in ccRCC also was higher than those in non-ccRCC type KCs lacking the high cytoplasmic lipid (chromophobe and papillary KC) (Fig. 5C). Along with that, higher NNMT expression positively correlated with shorter survival in ccRCC patients, but not in non-ccRCC type KCs (Fig. S5E). Experimentally, the levels of NNMT protein (Fig. 5D) and its product (1MNA; Fig. 5E) were significantly lower in the GPX8-lacking cells. In comparison, the level of NAD^+^, which increases upon NNMT inhibition and activates AMPK [34], was higher in GPX8-KO cells (Fig. 5E). Treatment of nicotinamide riboside (NR), a well-known cellular NAD^+^ generator, activated AMPK concentration-dependently (Fig. 5F). Correspondingly, a lower level of NAD^+^ in ccRCC tissues than in matched normal tissues was observed in our analysis of previous metabolomic data [35] (Fig. 5G). These results and findings seem to implicate NNMT’s involvement in GPX8’s negative regulation of AMPK via NAD^+^ modulation, which theory was further tested genetically. Knockdown of NNMT (shNNMT) metabolically led to 1MNA decrease, NAD^+^ increase (Fig. 5H), and AMPK activation, as shown by the increase in pAMPK and pACC in Caki1 and 786O cells (Fig. 5I). Importantly, it phenocopied the metabolic changes mediated by AMPK in GPX8-KO cells. The shNNMT decreased the DNL in terms of fatty acids and triglycerides (Fig. 5J) and decreased lipid droplet formation (Fig. 5K) without lipid uptake (Fig. S5F). Furthermore, cell growth was slower in the shNNMT ccRCC cell lines (Fig. 5L), and an NNMT inhibitor (6-methoxynicotinamide, 6MNA) inhibited ccRCC cells with concomitant activation of AMPK (Figs. S5G and H). These data indicate that NNMT mediates GPX8’s inhibitory effects on AMPK in suppressing lipogenesis and ccRCC cell survival.

### NNMT expression rescues GPX8-KO phenotype

**Fig. 6:**
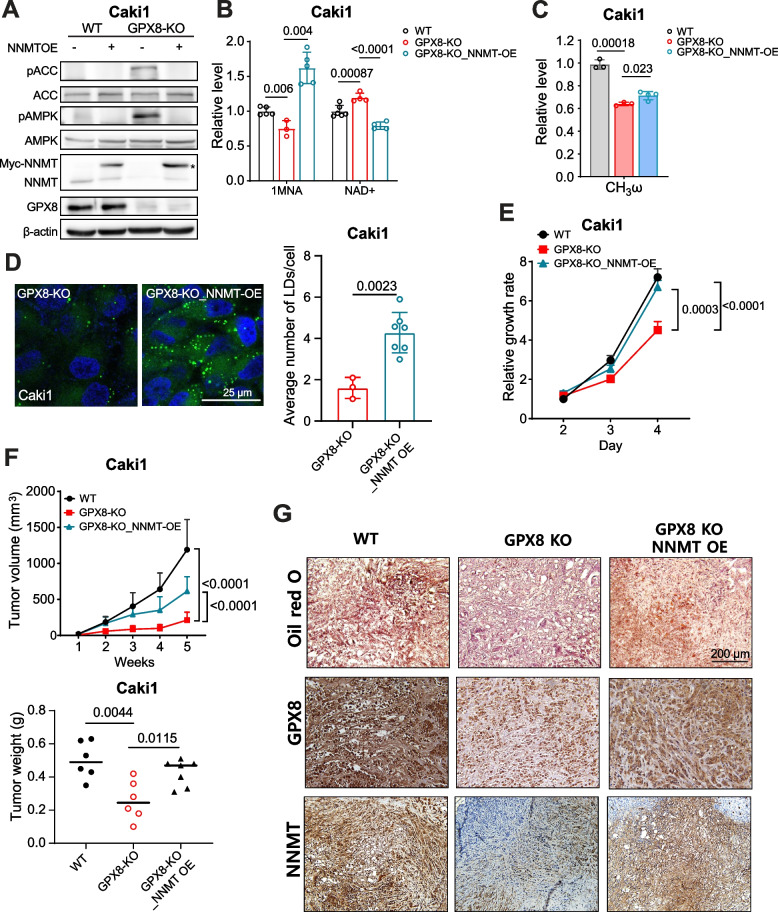
NNMT expression rescues the GPX8-KO phenotype. A-C, Western blot analysis of total and phosphorylated forms of ACC, AMPK, and NNMT, as normalized by β-actin (A), relative levels of 1MNA, NAD^+^ as measured by LC–MS/MS (B), relative levels of FA de novo synthesis as measured by NMR (C) in WT, GPX8-KO, and GPX8-KO with NNMT overexpression (OE) Caki1 cells. Data from (B) and (C) were normalized by total protein level. Asterisk indicates exogenous NNMT (Myc-tagged NNMT) expression in (A). D, Representative pictures (left) of neutral lipid BODIPY 493/503 from GPX8-KO Caki1 cells with and without NNMT OE. Quantitation of the lipid droplet (right) (*n* ≥ 3) as in Fig. 3F. E–F, Relative in vitro growth rates (E) and tumor growth (top) and tumor weight (bottom) of in vivo tumor xenograft in nude mice (*n* = 6–7) (F) for WT, GPX8-KO, and GPX8-KO with NNMT overexpression (OE) Caki1 cells. The experiment was performed three months after that in Fig. 2E and batch effects, i.e., initial lag phases for all the groups, may be present and the absolute values may be different from those in Fig. 2E. Data presented in panels (B), (C), (D), (E), and (F) are means ± SD (*n* ≥ 3). *P*-values were determined by unpaired *t*-test (B), (C), (D), and (F) (bottom) or two-way ANOVA with Geisser–Greenhouse correction (E) and (F) (top). G. Representative pictures of lipid contents (top) (Oil red O staining), GPX8 expression (middle), and NNMT expression (bottom) (IHC staining) from in vivo xenograft tumor tissues in nude mice with WT, GPX8-KO, and GPX8-KO with NNMT overexpression (OE) Caki1 cell transplantation; magnification, X200. For (A) through (G), the WT did not exhibit any appreciable difference from vector control (pCMV6-Entry2- Myc-DDK tag) in terms of GPX8 expression, cell viability, colony formation, and lipid droplet formation (Figs. S7A-D)

To confirm NNMT as a link between GPX8 and AMPK, we ectopically expressed NNMT in GPX8-KO cells. NNMT expression suppressed AMPK activation (Fig. 6A) and restored the 1MNA level while decreasing the NAD^+^ level (Fig. 6B). The functional lipogenic phenotype was also rescued relative to the GPX8-KO cells, as shown by the significantly restored DNL (Fig. 6C) and higher number of lipid droplets without higher lipid uptake levels (Figs. 6D and S6A). These data showed that NNMT expression phenocopies AMPK inhibition. NNMT expression also rescued cell survival (Fig. 6E), cell migration, and colony formation in GPX8-KO cells (Figs. S6B and C). This was recapitulated in an in vivo xenograft setting, where tumor growth inhibition was significantly lifted by the NNMT introduction (Figs. 6F and S6D). We also observed lower levels of GPX8, NNMT, and phenotypic lipid staining in the tumors from the GPX8-KO Caki1-bearing mice than those from the WT-xenografted ones (Fig. 6G). Furthermore, the lipid and NNMT levels were restored in the tumors from GPX8-KO/NNMT Caki1-bearing mice. Taken together, these data verify that NNMT is a mediator of GPX8’s suppression of ccRCC growth via AMPK inhibition.

### GPX8 modulates the NNMT expression through IL6-STAT3 signaling

**Fig. 7:**
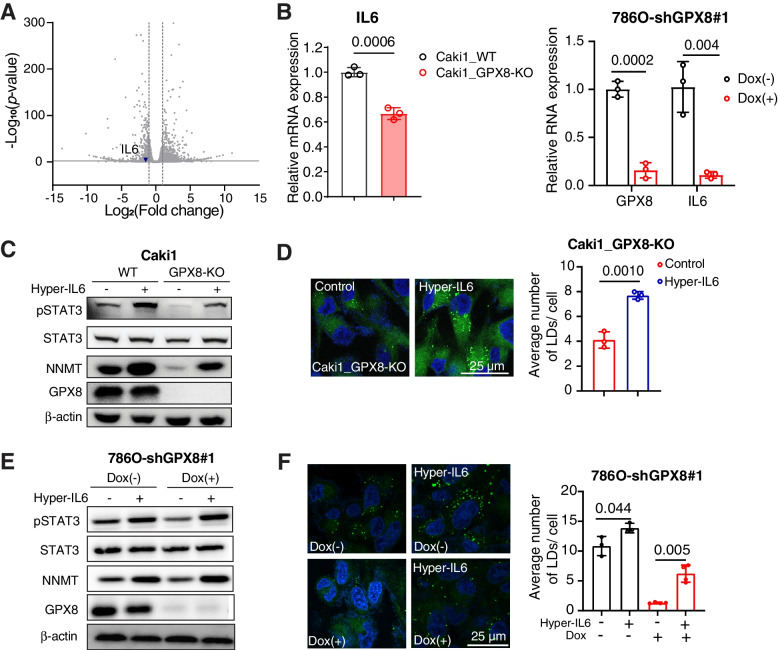
GPX8 modulates the NNMT expression through IL6-STAT3 signaling. A, Volcano plot for IL6 expression from our RNA-seq data. B, mRNA expression of IL6 by RT-qPCR comparing WT vs. GPX8-KO Caki1 cells (left) and shGPX8 786O with or without doxycycline (100 ng/mL) for 3 days (right). C, Western blot analysis of total and phosphorylated form of STAT3 (Ser 727), NNMT, and GPX8 normalized by β-actin from WT vs. GPX8-KO Caki1 with and without hyper-IL6 treatment (50 ng/mL) for 3 days. D, Representative picture (left) of neutral lipid BODIPY 493/503 staining of GPX8-KO Caki1 with and without hyper-IL6 treatment with same condition as in (C). Quantitation of the lipid droplet (right) (*n* = 3) as in Fig. 3F. E, shGPX8 786O cells were incubated with or without doxycycline (100 ng/mL) for 3 days before incubation with or without Hyper-IL6 (50 ng/mL) for 2 days. Western blot analysis of total and phosphorylated forms of STAT3 (Ser 727), NNMT, GPX8, and β-actin. F, Representative pictures (left) of neutral lipid BODIPY 493/503 staining of shGPX8 786O with or without Hyper-IL6 treatment with the same condition as in (E). Quantitation of the lipid droplet (right) (*n* ≥ 3) as in Fig. 3F. Data presented in panels (B), (D) and (F) are means ± SD (*n* ≥ 3). *P*-values were determined by unpaired *t*-test

We next asked how GPX8 regulates the NNMT expression in ccRCC cells. In separate studies, GPX8-KO suppressed IL6-STAT3 pathway in breast cancer cells [36], and IL6-STAT3 was shown to regulate NNMT expression [37]. However, this tripartite axis has not been demonstrated to act in concert in one system. We, therefore, hypothesized that GPX8-KO may suppress the IL6-STAT3 pathway in ccRCC cells, leading to the NNMT downregulation. As assumed, IL6 mRNA expression was lower in GPX8-KO than WT Caki1 cells (Figs. 7A and B), with concomitant lower phosphorylated STAT3 (pSTAT3 (Ser 727)) (Fig. 7C). Hyper-IL6, a combined form of IL6 and IL6 receptor used to activate IL6 signaling [38], not only recovered the pSTAT3 level but also rescued the NNMT expression (Fig. 7C). Furthermore, hyper-IL6 enhanced the phenotypic lipid accumulation in GPX8-KO Caki1 cell line (Fig. 7D). These results were consistent in shGPX8 786O cells (Figs. 7B, E and F). These data indicate that IL6-STAT3 mediates the GPX8 regulation of NNMT.

### GPX8-NNMT axis is independent of VHL and regulated by ROS

**Fig. 8:**
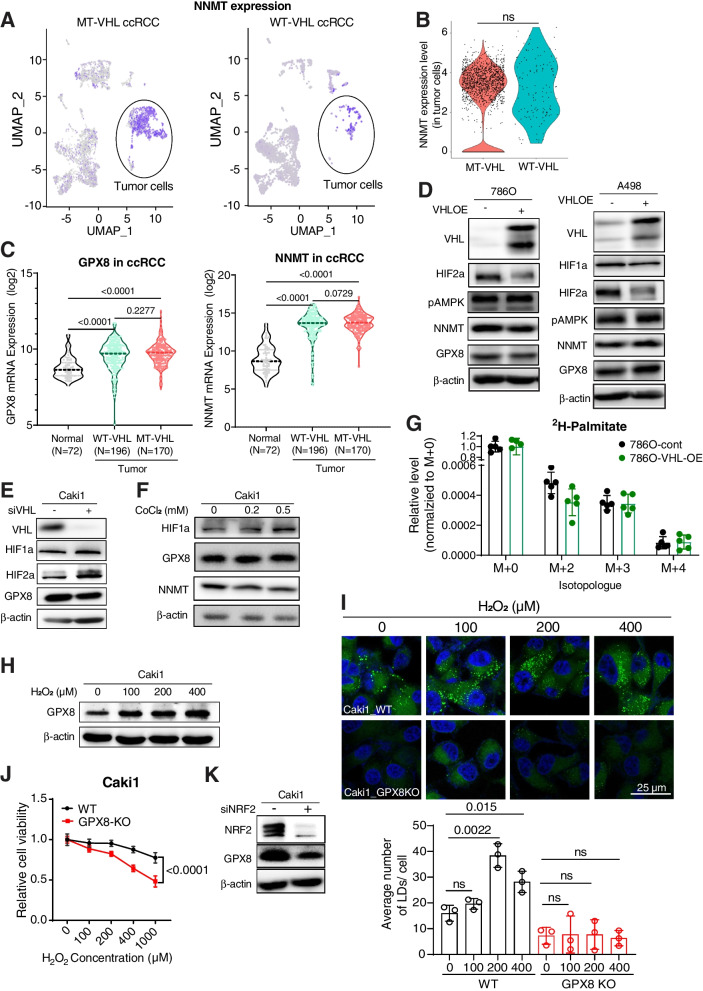
GPX8-NNMT axis is independent of VHL mutation status and regulated by ROS. A, Single-cell RNA-seq data for ccRCC tumors with WT-VHL and MT-VHL. Tumor cells are indicated with black ellipses. B, NNMT expression levels in tumor cells from (A). C, mRNA expression levels of GPX8 (left) and NNMT (right) according to normal and VHL status of tumors in ccRCC patients. The mRNA expression values were obtained from the TCGA-KIRC dataset. *P*-values were determined by Mann–Whitney U test. D-F, Western blot analysis of VHL, HIFs, pAMPK α1 (T183), α2 (T172), GPX8, and NNMT upon ectopic expression of VHL in 786O and A498 cells, or VHL siRNA and CoCl2 treatment in Caki1 cells. G, Total DNL measurement by incorporation of deuterium from 10% D_2_O for 2 days. The fractions of M + 2, M + 3, and M + 4 normalized by M + 0 fraction of palmitate, as measured with LC–MS, from 786O cells with or without VHL-OE. H-J, The effects of treatment of serial concentrations of H_2_O_2_ to WT and GPX8-KO Caki1. Western blot analysis of GPX8 upon 1-h incubation (H), representative pictures of neutral lipid staining (top) upon 6-h treatment and quantitation of the lipid droplet (bottom) (*n* = 3) as in Fig. 3F (I), and relative cell viability upon 24-h treatment (J). K, Western blot analysis of NRF2 and GPX8 from Caki1 upon treatment of scrambled or NRF2 siRNA. Data presented in (G), (I), and (J) are means ± SD (*n* ≥ 3). *P*-value in (I) were determined by unpaired* t*-test. *P*-value in (J) was calculated by two-way ANOVA with Geisser Greenhouse correction. ns, not significant

To find the upstream regulator of the GPX8-NNMT axis, we first investigated its VHL-HIF-dependence, since VHL mutation is the most frequent in ccRCC, and HIF has been shown to upregulate GPX8 expression in HeLa cells [39]. When we analyzed the ccRCC single-cell RNA sequencing data [25], NNMT expression was clearly higher in tumor cells than in other cell types but similar between the WT- and MT-VHL tumor cells (Figs. 8A, B, and S8A) (Unfortunately, GPX8 expression could not be analyzed, due to non-reliable detection in this particular dataset). Also, neither GPX8 nor NNMT exhibited different expression levels in ccRCC tissues with or without VHL mutation in the TCGA dataset (Fig. 8C). Consistent with this VHL-independence, GPX8-NNMT expression was not affected either by VHL reconstitution/HIF downregulation in VHL-negative cells (786O and A498) or by HIF activation through VHL siRNA or CoCl_2_ treatment in VHL-WT cells (Caki1) (Figs. 8D-F). These protein level expressions were consistently observed at mRNA levels for GPX8-NNMT (Fig. S8B). The absence of VHL effect on GPX8 and NNMT expression was also observed regardless of time points of experiment (Fig. S8C). The VHL independence was also observed at the AMPK level, which showed that AMPK activation is also independent of VHL expression status (Figs. 8D and S8C). This is also corroborated by patient level data showing no significant difference in pAMPK (T172) levels between VHL wild type (*n* = 160) and mutant patients (*n* = 154) from the TCGA database (Fig. S8D). For a direct metabolic effect, DNL flux, measured by ^2^H incorporation into fatty acids in D_2_O, was not affected by VHL restoration in VHL-negative ccRCC cells (786O) (Fig. 8G). These data indicate that the GPX8-NNMT axis and the high DNL activity in ccRCC are not regulated by VHL. As GPX’s main functions are related to oxidative stress, and acute reactive-oxygen-species (ROS) treatment induces lipid droplet accumulation [40], we tested ROS as a possible GPX8 regulator. H_2_O_2_, an upstream trigger of GPX8 upregulation in brain cells [41], enhanced GPX8 expression in ccRCC cells concentration dependently (Fig. 8H); meanwhile, it also gradually increased phenotypic lipid accumulation in GPX8-WT cells, but not in GPX8-KO cells (Fig. 8I). In addition, the presence of GPX8 protected the ccRCC cells from cell death by high H_2_O_2_ concentration (Fig. 8J). Consistently in this regard, siRNA of NRF2, a key mediator of cellular ROS responses induced by H_2_O_2_, reduced GPX8 expression (Fig. 8K). These results show that H_2_O_2_ may be correlated with GPX8 and lipid accumulation, but proof of the causality requires significantly more data.

## Discussion

The “clear cell” phenotype with high lipid content as in ccRCC is rare in cancers of other major organs such as the liver, brain, lung, and breast. However, ccRCC is by far the major subtype in KC, and therefore, there has been substantial interest in how this high lipid content arises in ccRCC. Theoretically, high lipid level can be due to several factors including high DNL, low lipid degradation, high lipid uptake, or high lipid storage processes. Indeed, there have been reports implicating the roles of low FAO (lipid degradation), lipid storage, or unsaturated lipid uptake in ccRCC tumorigenesis. Du et al*.* suggested that low FAO due to CPT1A inhibition by activated VHL-HIF signaling is the main driver of lipid increase and a necessary process for tumor growth in ccRCC [11]. Interestingly though, in the same study, fatty acid uptake was not affected by VHL status and did not contribute to the observed lipogenic phenotype. For lipid storage, PLIN2 involved in transporting lipids into lipid droplets has been shown to be induced by HIF2α, which protects cancer cells from ER stress [9]. In addition, storage of serum-derived oleate to triacylglycerol in lipid droplets has been proposed to counter increased saturated fatty acids under hypoxia, which condition may be toxic to cells [14]. As for high lipid synthesis, a few reports have suggested its possible involvement in ccRCC. For example, VHL-regulated ACLY, which provides acetyl-CoA for lipid synthesis, was shown to be higher in ccRCC tumors [42]. However, this study did not show ACLY’s actual contribution to high lipid phenotype in ccRCC. In addition, AMPK and SREBP1, well-known mediators of lipid synthesis, were implicated in the growth and malignant phenotype of ccRCC [32, 43]. Still, important questions remain as to which upstream regulators/pathways are involved in controlling these known metabolic factors for lipid synthesis. Our finding of upstream regulators leading to AMPK, namely GPX8-IL6-STAT3-NNMT, should provide new insights into how DNL is regulated for its characteristic phenotype in ccRCC. It is interesting to note that the involvement of DNL in high lipid content is actually consistent with a previous result showing a glucose-concentration-dependent increase in lipid droplet accumulation in ccRCC cells [11].

An intriguing aspect of the GPX8-NNMT axis for DNL is its independence of VHL mutation, the most frequent genetic alteration in ccRCC. Of note, previous studies on decreased FAO [10, 11] and enhanced lipid storage [9] for high lipid contents in ccRCC all reported VHL-dependence of those processes. For one thing, these studies used the expression level or activity of CPT1A as the indication of FAO. Although CPT1A is a much-used marker as the rate-limiting step of FAO, other enzymes in actual beta oxidation steps, i.e., keto acyl thiolase, could also be a limiting factor [44], particularly in ccRCC [32]. In this context, we used the isotope-tracing method to directly test VHL independence of DNL as driven by GPX8-NNMT. For another, we showed that expressions of GPX8 and NNMT are not affected by heterologous expression of VHL in MT-VHL ccRCC cells or by knockdown of VHL in WT-VHL ccRCC cells. In addition, both WT and MT-VHL cell lines required DNL for lipid droplet formation and cell growth. In line with our finding, a previous study on SREBP1-mediated lipogenesis in ccRCC found it to be independent of VHL mutation [45]. Moreover, activation of the VHL-HIF pathway or HIF does not seem to be correlated with high DNL in other models. For example, VHL-KO mice with activated HIF2α in the liver exhibited lower lipogenic gene expression with impaired FAO [46], and HIF1α expression correlated negatively with ACC1 in human adipose tissue [47]. It is worth noting that, along with VHL mutation, there are several mutually mutated genes such as PBRM1, BAP1, and SETD2 in the most frequently lost chromosome  3p in ccRCC [48], which fact demonstrates the complicated relationship among contributors to ccRCC. As high lipid content is characteristic of ccRCC tissue regardless of VHL mutation status, the proposed VHL-independence of GPX8-NNMT axis-mediated DNL is not mutually exclusive to the previously reported VHL-dependent FAO in ccRCC. The former may operate in ccRCC with both VHL-WT and VHL-MT status, while the latter may be relevant mainly in those with VHL-mutation. In this regard, it will be an interesting topic for future studies to see the effects of GPX8-NNMT axis on FAO and the involvement of VHL.

As suggested by the name and ER localization, GPX8’s basic functions are related to ER stress relief and H_2_O_2_ neutralization. Our data and previous reports have shown that GPX8 is induced by H_2_O_2_ [41] and that loss of GPX8 can cause imbalanced lipid composition in ER [20] and ER-stress-induced cell death [16]. As regulation of ER stress and H_2_O_2_ is critical for rapid proliferation and survival of cancer cells, GPX8 seems to be an important factor linking these basic cancer cell features to the lipogenic phenotype of ccRCC. Although our data showed that H_2_O_2_ induced GPX8 and lipid accumulation, we do not exclude the possibility that H_2_O_2_ may act through other pathways for lipid regulation. H_2_O_2_ has a pleiotropic activity affecting several competing pathways [49, 50]. In this sense, H_2_O_2_ as a potential upstream regulator of GPX8-IL6-STAT3-NNMT axis for DNL may need to be further tested in several different contexts. It is worth noting that sunitinib-resistance was reported to be mediated by ER-stress response in ccRCC [51]. The tyrosine kinase inhibitor was shown to activate PERK, a key signal mediator during ER-stress, and to upregulate proinflammatory cytokine such as IL6, IL8 and TNF-alpha [51]. As H_2_O_2_ is an ER-stress inducer and GPX8 is related to ER-stress and IL6, the roles of GPX8 in drug-resistance in ccRCC may be an interesting future study topic. In contrast to the closely related GPX7, which has been posited as a tumor suppressor [52], emerging evidence has implicated GPX8 as a pro-tumorigenic factor in various cancers such as gastric, lung, and breast cancer [21, 22, 36]. Nevertheless, it should be noted that enhanced lipid synthesis, shown in our study, may not always be associated with GPX8 in other tissues that express higher GPX8 in tumors than in normal tissues. For example, lipid synthesis is dispensable in primary breast cancer [53], and FAO, generally exclusive to fatty acid synthesis, seems required for the growth of glioblastoma [54] or liver cancer cells [55]. In fact, the protumorigenic property of GPX8 does not seem to be universal either, considering that its promotor region has HIF-response elements whose activation leads to the inhibition of proliferation through suppression of AKT activation in HeLa cells [39]. Tissue-specific functions of GPX8 have also been noted in non-cancer settings. For example, covalent binding between GPX8 and caspase 4 inhibited inflammation associated with colitis [18], and GPX8 was involved in protection from bleomycin-induced lung injury in IGF1R knockout animals [56]. Therefore, GPX8, despite its general involvement in H_2_O_2_ and ER stress, has downstream functions that can substantially differ according to tissues and pathophysiological conditions, and its lipid metabolism-associated roles may be uniquely related to the characteristic lipogenic phenotype in ccRCC.

Several reports have addressed NNMT’s involvement in lipid metabolism in non-cancer settings, though the issue remains controversial. For the liver, NNMT was shown to decrease hepatic lipogenic gene expression and to improve systemic lipid parameters [10], which findings were supported by a later study [57]. However, another study found that high NNMT expression causes fatty liver disease by modulating the NAD level that, in turn, regulates SIRT3 [58]. Similar phenotypes were found in adipose tissues, where NNMT was associated with diet-induced obesity by its regulation of energy expenditure through NAD, the polyamine pathway, and histone modification [59]. For cancer, NNMT was primarily implicated in poor prognosis or metastasis in various tumors such as pancreatic, gastric, ovarian, breast cancer, and glioblastoma [60]. In ccRCC, too, NNMT was suggested to be a cancer marker and contributor to metastasis [61]. Still, upstream regulators of NNMT in cancer were shown to be diverse, as in BRCA1 for ovarian cancer, HNF1β for thyroid cancer, and SHH for pancreatic cancer [60]. In liver cancer, unlike other cancers, the NNMT level is lower than in normal tissues [62], and in fact its downregulation has been implicated in liver cancer [63]. These studies in both normal and cancer settings posit that NNMT, just like its upstream-GPX8, has context- and tissue-dependent roles. Despite this considerable literature describing the roles of NNMT in cancer and lipogenesis in non-cancer diseases, there is a surprising lack of studies on possible links between NNMT and lipogenesis in cancer. Therefore, NNMT’s inhibition of AMPK leading to DNL is an important new finding in ccRCC, as reflects its critical characteristic phenotype. These findings are also consistent with a recent study on alcoholic fatty liver disease, where NNMT was activated by ER stress to induce lipogenesis [34]. Indeed, obesity increases the risk of ccRCC, and a recent study concluded that chemerin, an adipokine from ccRCC cells, is associated with the adipocyte-like phenotype of ccRCC [10]. With NNMT as a linker between DNL and ccRCC along with NAD metabolism as a well-established factor in obesity, NNMT and NAD may be additional molecular clues to the intriguing relationship between obesity and ccRCC.

## Conclusion

In conclusion, we identified the GPX8-NNMT axis that controls DNL for the characteristic phenotype of high lipid content and survival of ccRCC. As GPX8-KO exhibits no apparent phenotype under standard conditions [18], this axis could be targeted as a new therapeutic approach to ccRCC. Despite the consistency of our results for the GPX8-NNMT axis, there might be still-unknown upstream/intermediate pathways that could not be addressed in this study, considering the diverse regulators of GPX8 and NNMT in various conditions and tissues, warranting further investigations.

## Supplementary Information

**Additional file 1:**

## Data Availability

The RNA sequencing data was deposited in GEO database GSE193249. Supplementary materials: including key resources table and eight supplementary figures.
