# Supplementary material for: GPX8 regulates clear cell renal cell carcinoma tumorigenesis through promoting lipogenesis by NNMT
Source: J Exp Clin Cancer Res. 2023 Feb 7;42:42. doi: 10.1186/s13046-023-02607-2 (PMC9903620; doi:10.1186/s13046-023-02607-2)
Supplement: Supplementary file 1 — Additional file 1: [file 13046_2023_2607_MOESM1_ESM.docx]

## Supplementary information

**GPX8 regulates clear cell renal cell carcinoma tumorigenesis through promoting lipogenesis by NNMT**

Tin Tin Manh Nguyen^1†^, Thi Ha Nguyen ^1†^, Han Sun Kim^1^, Thien T.P. Dao^1^, Yechan Moon^1^, Munjun Seo^1^, Sunmi Kang^1^, Van-Hieu Mai^1, 2^, Yong Jin An^1^, Cho-Rok Jung^3,4^, Jin-Mo Kim^1*^, Sunghyouk Park^1*^

^1^, Natural Products Research Institute, College of Pharmacy, Seoul National University, Seoul 08826, Republic of Korea.

^2^, Molecular Biology Department, School of Medicine, Vietnam National University, Ho Chi Minh City 70000, Vietnam.

^3^, Korea Research Institute of Bioscience and Biotechnology (KRIBB), Daejeon 34141, Republic of Korea.

^4^, Department of Functional Genomics, Korea University of Science and Technology (UST), Daejeon 34113, Republic of Korea.

^†^These authors contributed equally to this work.

*Correspondence:

Jin-Mo Kim

[jmk0831@snu.ac.kr](mailto:jmk0831@snu.ac.kr)

Sunghyouk Park

[psh@snu.ac.kr](mailto:psh@snu.ac.kr)

Keywords: Clear cell renal cell carcinoma (ccRCC), GPX8, NNMT, AMPK, *de novo* lipogenesis (DNL)

# SUPPLEMENTARY MATERIALS:

# *Key resources table*

| **Reagent or resource** | **Source** | **Identifier, Catalogue** |
| --- | --- | --- |
| **Antibody** | | |
| GPX8 Rabbit pAb | Abcam | ab183664 |
| GPX8 Rabbit pAb | MyBioSource (San Diego, CA, USA). | MBS7005948 |
| β-actin Mouse mAb | Santa Cruz | sc-47778 |
| Phospho Acetyl-CoA Carboxylase (Ser79) (pACC) Rabbit pAb | Cell signaling | #3661 |
| Acetyl-CoA Carboxylase (ACC) Rabbit pAb | Cell signaling | #3662 |
| Phospho AMPK alpha 1 (T183) + AMPK alpha 2 (T172) Rabbit pAb | Abcam | ab23875 |
| AMPK alpha 1 + AMPK alpha 2 Mouse mAb | Abcam | ab80039 |
| NNMT Mouse mAb | Santa Cruz | sc-376048 |
| STAT3 Rabbit pAb | Cell signaling | #12640 |
| Phospho STAT3 (Ser727) (pSTAT3) Rabbit pAb | Cell signaling | #9134 |
| VHL Rabbit pAb | Cell signaling | #68547 |
| HIF1α Rabbit pAb | Bethyl | A300-286A |
| HIF2α Rabbit pAb | Abclonal | A7553 |
| **Chemicals** | | |
| U^13^C-glucose | Cambridge Isotope Laboratories, Inc. | CLM-1396-PK |
| U^13^C-palmitate | Cambridge Isotope Laboratories, Inc. | CLM-3943-0.5 |
| Deuterium oxide (D_2_O) | Cambridge Isotope Laboratories, Inc. | DLM-4-99-1000 |
| Bodipy 493/503 | ThermoFisher Scientific | D3922 |
| Bodipy 500/510 C1, C12 | ThermoFisher Scientific | D3822 |
| TOFA | medchemexpress | HY-101068 |
| C75 | Cayman | Q-10005270 |
| AICAR (5-Aminoimidazole-4-carboxamide ribonucleotide) | medchemexpress | HY-13417 |
| 6MNA (6-Methoxynicotinamide)- JBSNF-000088 | Chemcruz | sc-300024 |
| Compound C (Dorsomorphin) HCl | medchemexpress | HY-13418 |
| NR (Nicotinamide riboside) | medchemexpress | HY-123033 |
| Hyper-IL6 (Recombinant Human IL-6/IL-6R alpha Protein Chimera) | R&D Systems, Inc. | 8954-SR-025/CF |
| Matrigel | BD Biosciences, San Jose, CA | 356234 |
| **Commercial assays** | | |
| CCK8 (Cell Counting Kit-8) | Dojindo | CK04-11 |
| chemiluminescent reagent ECL Solution | Abfrontier WESTSAVE | Cat# F-QC0106 |
| SYBR green-based detection (iTaq TM Universal SYBR Green Supermix) | Bio-Rad, USA | Cat# 172-5120 |
| High-Capacity cDNA Reverse Transcription Kit | Applied Biosystems, Inc., USA | Cat# 4368814 |
| **Deposited data** | | |
| RNA seq | GSE193249 | NA |
| **Experimental models: cell lines** |  |  |
| Caki1 | Korean Cell Line Bank | NA |
| 786O | ATCC | NA |
| A498 | Korean Cell Line Bank | NA |
| HEK293T | Prof. Sang-Min Jeon | NA |
| **Experimental models** | | |
| Tissue array of kidney carcinoma and matched adjacent tissue | US Biomax, Inc., Derwood, MD, USA | KD482 |
| Male BALB/c nude mice | Orient Bio Laboratory Animal Research Center Co., Ltd (Seoul, Korea) | NA |
| **Oligonucleotides** | | |
| IL6 | Bioneer | P211161 |
| NNMT | Bioneer | P270099 V |
| GPX8 | Bioneer | F: CCTCAAGAATGCCAGATGAGTG  R: AGTAAGTGTGTTAATTGACTTTTAGAGTTG |
| GAPDH | Bioneer | F: GAGTCAACGGATTTGGTCGT  R: TTGATTTTGGAGGGATCTCG |
| β-actin | Bioneer | F: GGACTTCGAGCAAGAGATGG  R: AGCACTGTGTTGGCGTACAG |
| **Lentivirus or recombinant DNA** | | |
| pSpU6Cas9-2A-Puro for GPX8 | Macrogen | sgGPX8 (AGCTGCAAGAGGCTCCATGTTGG) |
| shNNMT#1, #2, control | Genecopoeia (US) | HSH011860-LVRU6H |
| pCMV6-Entry-Myc-DDK-tag carried NNMT | Origene | CAT#: RC200641 |
| pCMV6-Entry-Myc-DDK-tag carried GPX8 | Origene | CAT#: RC205924 |
| pCMV6-Entry-Myc-DDK-tag carried VHL | Origene | CAT#: RC216151 |
| pCMV6-Entry Mammalian Expression Vector (control) | Prof. Sangkook Lee | CAT#: PS100001 |
| shRNA GPX8 (pLKO-TET-On) | NA | shGPX8#1  CCATGAGGGTTTGGTCTCATT  shGPX8#2  GCCATTGCGTTTCTAATAGAA |
| siRNA AMPKα1 | Bioneer | siRNA No 5562-1(Human, PRKAA1) |
| siRNA AMPKα2 | Bioneer | siRNA No 5563-1(Human, PRKAA2) |
| siRNA VHL | Bioneer | siRNA No 7428-1(Human, VHL) |
| siRNA NRF2 | Bioneer | siRNA No 4780-1 (Human, NRF2) |
| **Software and algorithms** |  |  |
| Topspin | Bruker | Ver. 3.6.3 |
| R | NA | Ver. 4.1.1 |
| GraphPad Prism | NA | Ver. 9.3.0 |

# *Lead contact and material availability*

Further information and requests for resources and reagents should be directed to and will be fulfilled by the Lead Contact, Sunghyouk Park ([psh@snu.ac.kr](mailto:psh@snu.ac.kr)).

# SUPPLEMENTARY FIGURES


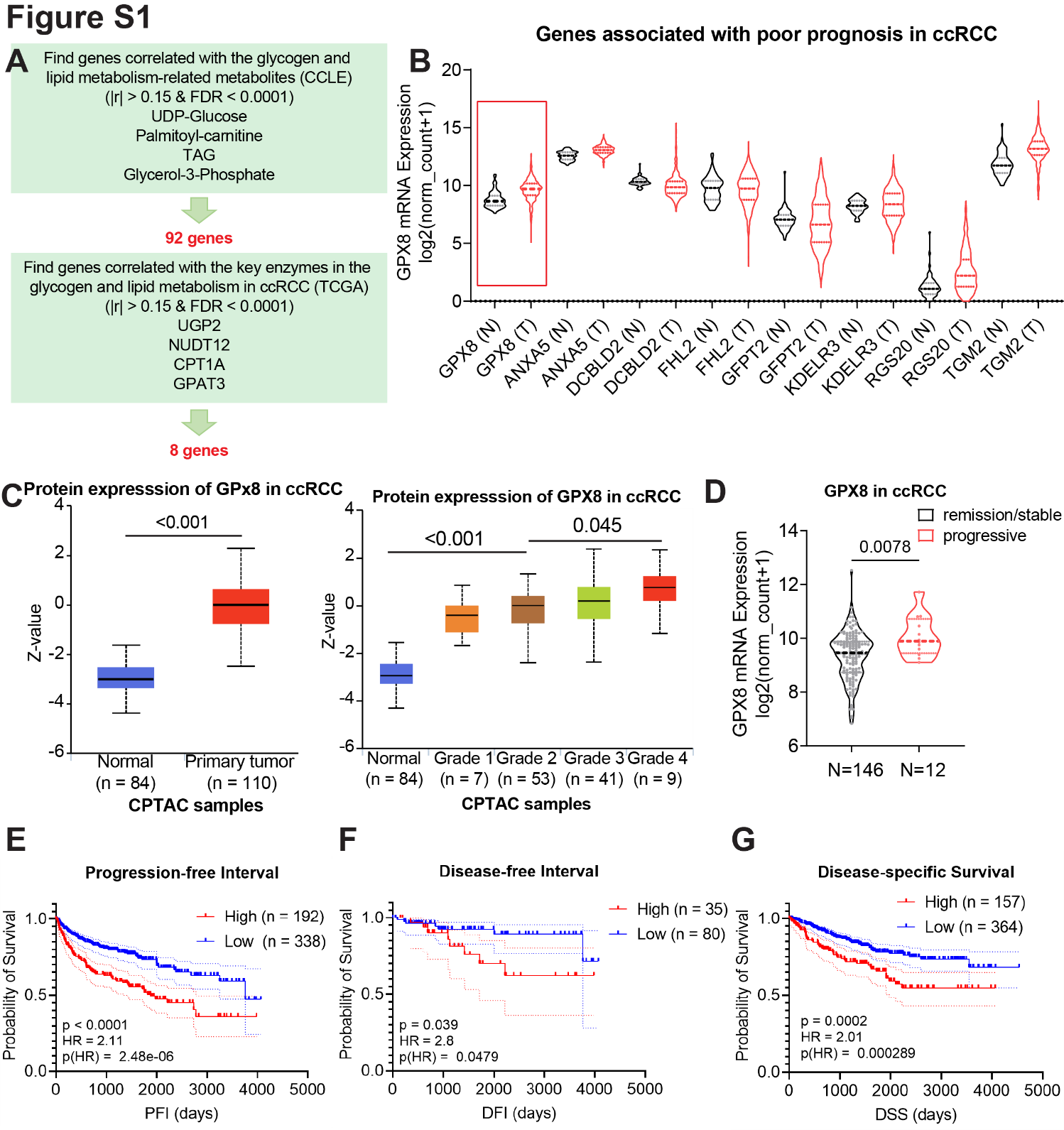
**Figure S1. (Related to Fig. 1) GPX8 is associated with higher grade and poor prognosis in ccRCC**
**A,** Scheme for screening to find genes correlated with key metabolites (top; CCLE database) and key genes (bottom; TCGA-KIRC database) for glycogen and lipid metabolism in ccRCC. **B,** The expression of candidate genes from (**A**) between tumor (T) vs. normal (N) tissues in ccRCC patients from the TCGA-KIRC dataset. **C,** Protein expression of GPX8 in tumor vs. normal (left) or according to the tumor grade (right) in ccRCC patients from the CPTAC dataset. **D,** Analysis of GPX8 mRNA expression in ccRCC patients with stable disease or remission vs. progressive disease after primary therapy. *P-*values were determined by Mann-Whitney U test. **E-G,** Progression-free interval (**E**), disease-free interval (**F**), and disease-specific survival plots (**G**) according to GPX8 mRNA expression from TCGA-KIRC database. See method for a detail description of analysis.


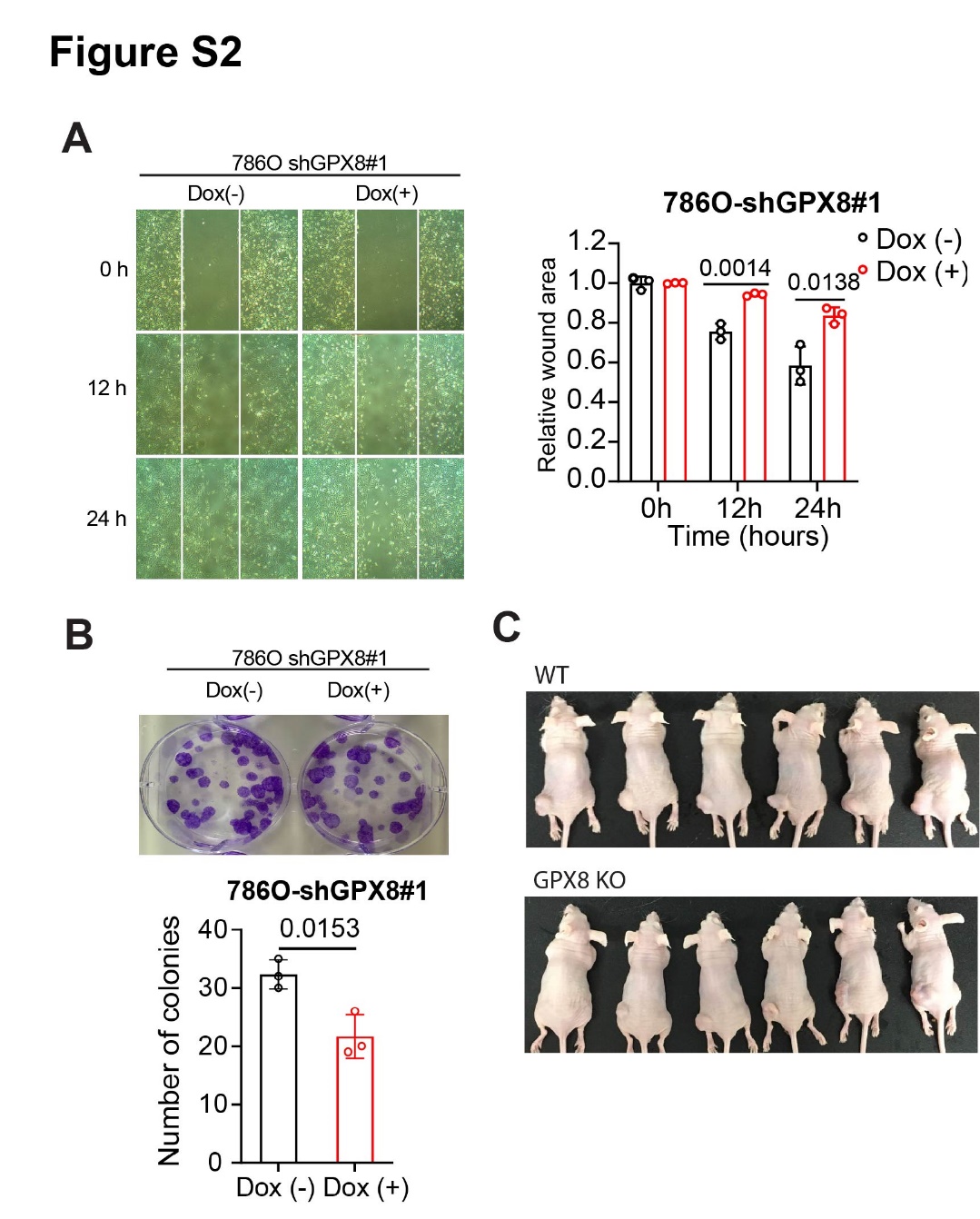


**Figure S2. (Related to Fig. 2) GPX8 is involved in ccRCC cell growth *in vitro* and *in vivo***

**A**, Scratch assay for 786O shGPX8 cells at 0, 24, and 48 h (left) that had been incubated with or without doxycycline 100 ng/mL for 3 days. Relative wound area for cells with and without doxycycline treatment (right) (*n* = 3). **B**, Clonogeneic assay for 786O shGPX8 cells with and without doxycycline treatment (100 ng/ml) after plating 100 cells in 6-well plate for 10 days (top) and bar graph for the number of colonies (bottom) (*n* = 3). Data presented in (**A**) and (**B**) panels are means ± SD (*n*=3). *P*-value was calculated by unpaired *t*-test. **C**. Photograph of tumors obtained on the last day of the 4^th^ week after implantation of WT or GPX8-KO Caki1 cells.


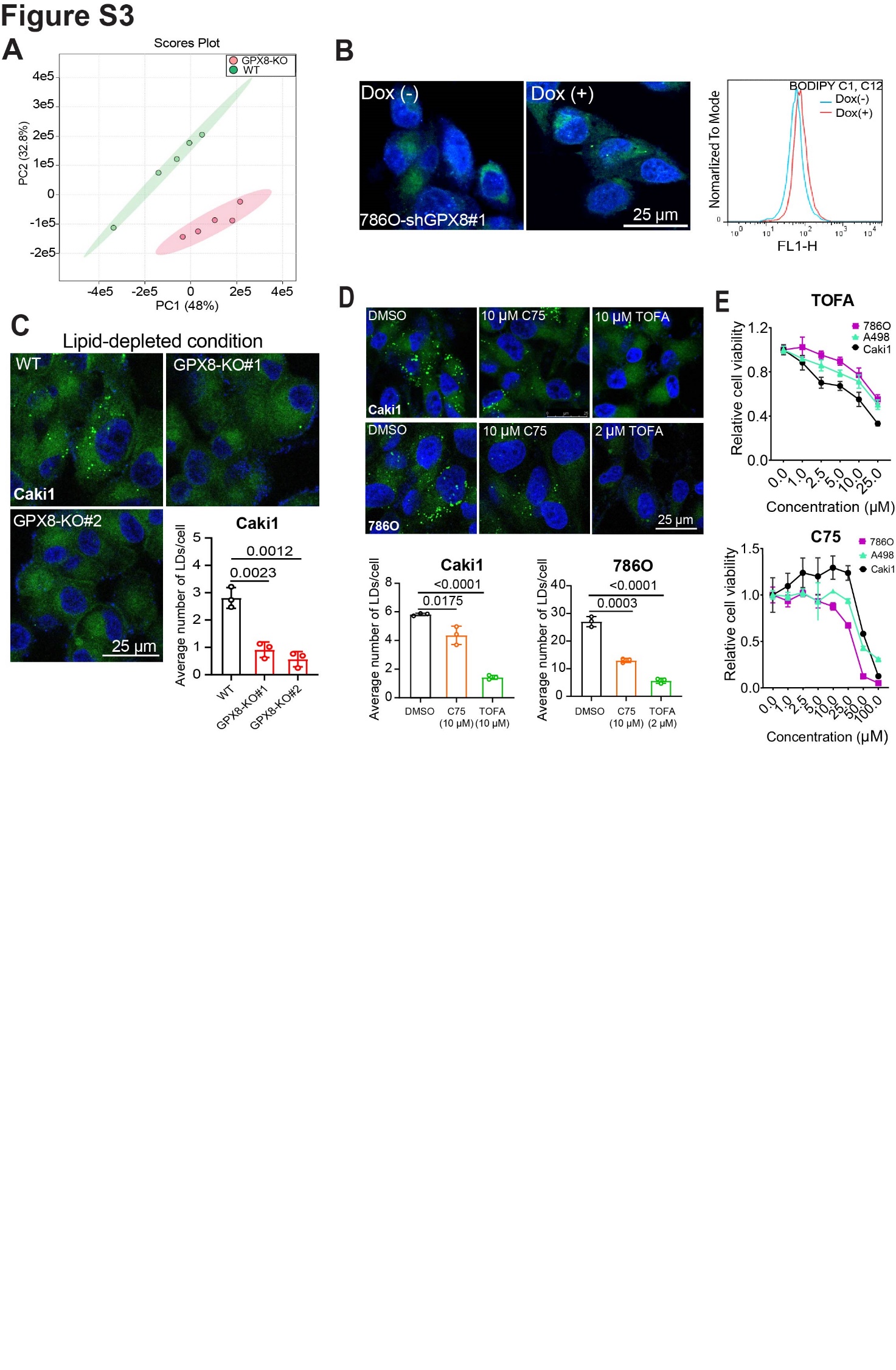


**Figure S3. (Related to Fig. 3) GPX8 regulates lipid metabolism in ccRCC**

**A,** Principal component analysis (PCA) from metabolomic data of WT vs. GPX8-KO Caki1 (*n* = 5). **B**, Representative pictures of BODIPY 500/510 C1, C12 staining 786O shGPX8 cells incubated with or without doxycycline (100 ng/mL) for 3 days (left). The fluorescent intensity of lipid uptake was also measured by flow cytometry (right). **C,** Representative pictures of neutral lipid BODIPY 493/503 staining in WT and GPX8-KO Caki1 growing in lipid-depleted medium. Quantification of lipid droplet as in Fig. **3F** (*n* = 3). **D,** Representative pictures (top) of neutral lipid BODIPY 493/503 staining of Caki1 and 786O cells treated with C75 and TOFA for 3 days vs. DMSO. Quantitation of the lipid droplet (bottom) (*n* = 3) as in Fig. **3F**. **E,** Relative cell viability of Caki1, 786O, and A498 cell lines treated with C75 and TOFA for 3 days. Data presented in panels (**A**), (**C**), (**D**) and (**E**) are means ± SD (*n* ≥ 3). *P*-value was calculated by unpaired *t*-test.


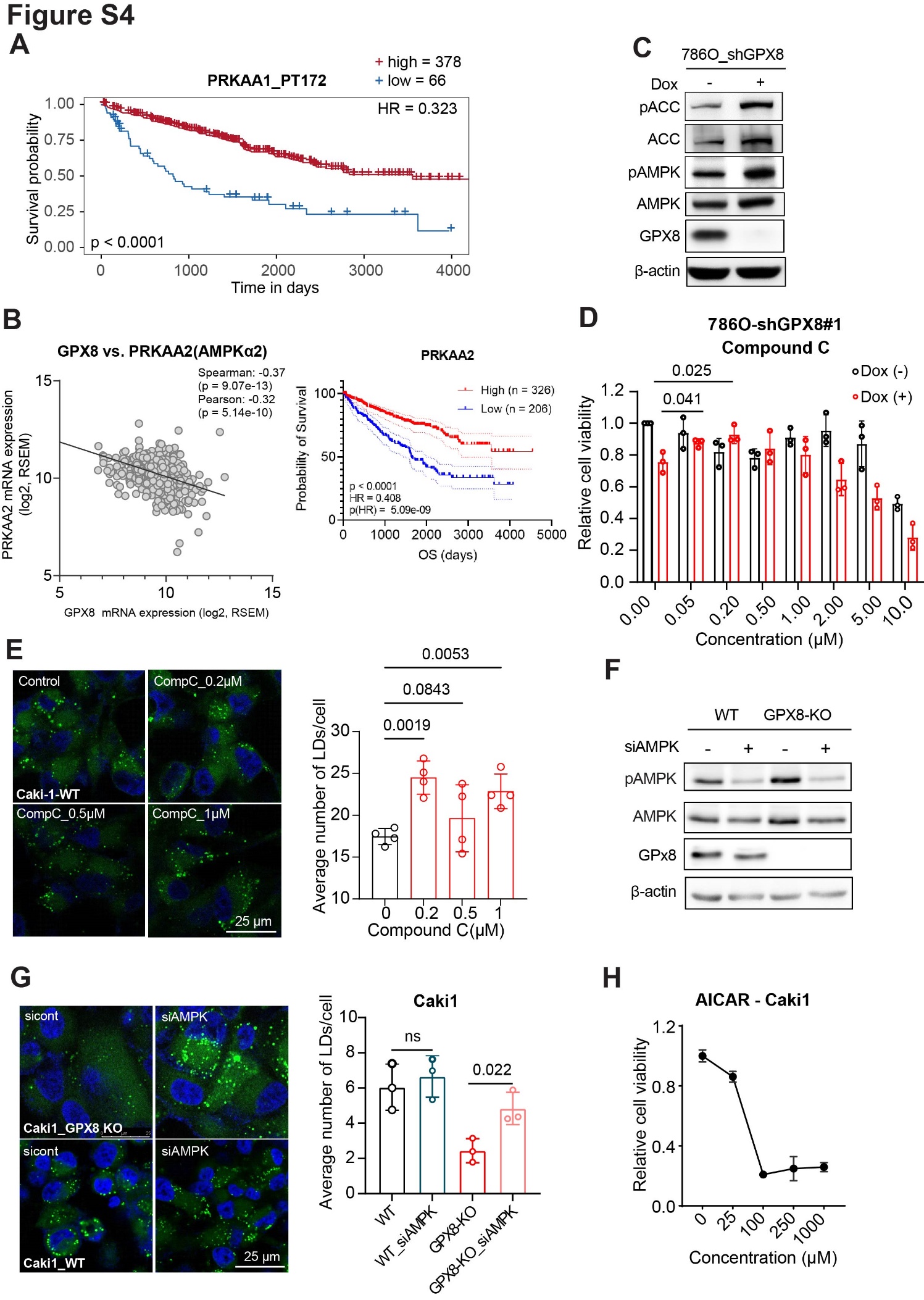


**Figure S4. (Related to Fig. 4) GPX8 enhances lipid accumulation by inhibiting AMPK**

**A,** Overall survival plot according to pT172 of AMPK for ccRCC patients from TCGA-KIRC analyzed by TRGAted (<https://nborcherding.shinyapps.io/TRGAted/>). **B,** Correlation between GPX8 and AMPKα2 (PRKAA2) mRNA expression (left) and overall survival plot according to AMPKα2 expression (right) for ccRCC patients from the TCGA-KIRC dataset. **C,** Western blot analysis of the phosphorylated and total forms of ACC and AMPK, normalized by β-actin in shGPX8 786O cells with or without doxycycline treatment. **D,** Cell viability for 786O cells upon compound C treatment. 786O shGPX8 cells were incubated with or without doxycycline (100 ng/mL) for 3 days. These cells then were treated with Compound C in a range of concentration for 2 days. **E**, Representative pictures (left) of neutral lipid BODIPY 493/503 staining in Caki1 treated with different concentrations of compound C. Quantitation of lipid droplet as in Fig. **3F** (right) (*n* = 4). **F,** Western blot analysis of the total AMPK and phosphorylated AMPK (AMPK α1 (T183) and AMPK α2 (T172)) from WT or GPX8 KO Caki1 cells treated with siAMPK or scrambled siRNA. **G,** Representative pictures (left) of neutral lipid BODIPY 493/503 staining of WT and GPX8 KO Caki1 cells at the same condition as in (**F**). Quantitation of lipid droplet as in Fig. **3F** (right) (*n*=3) (**H**), Cell viability of Caki1 cells treated with a serial concentration of AICAR for 3 days. Data presented in (**D**), (**E**), (**G**), and (**H**) are means ± SD (*n* ≥ 3). *P*-values in panels (**D**), (**E**), and (**G**) are calculated by unpaired *t*-test. *P*-value in (**H**) is calculated by two-way ANOVA with Geisser-Greenhouse correction. *ns*, not significant


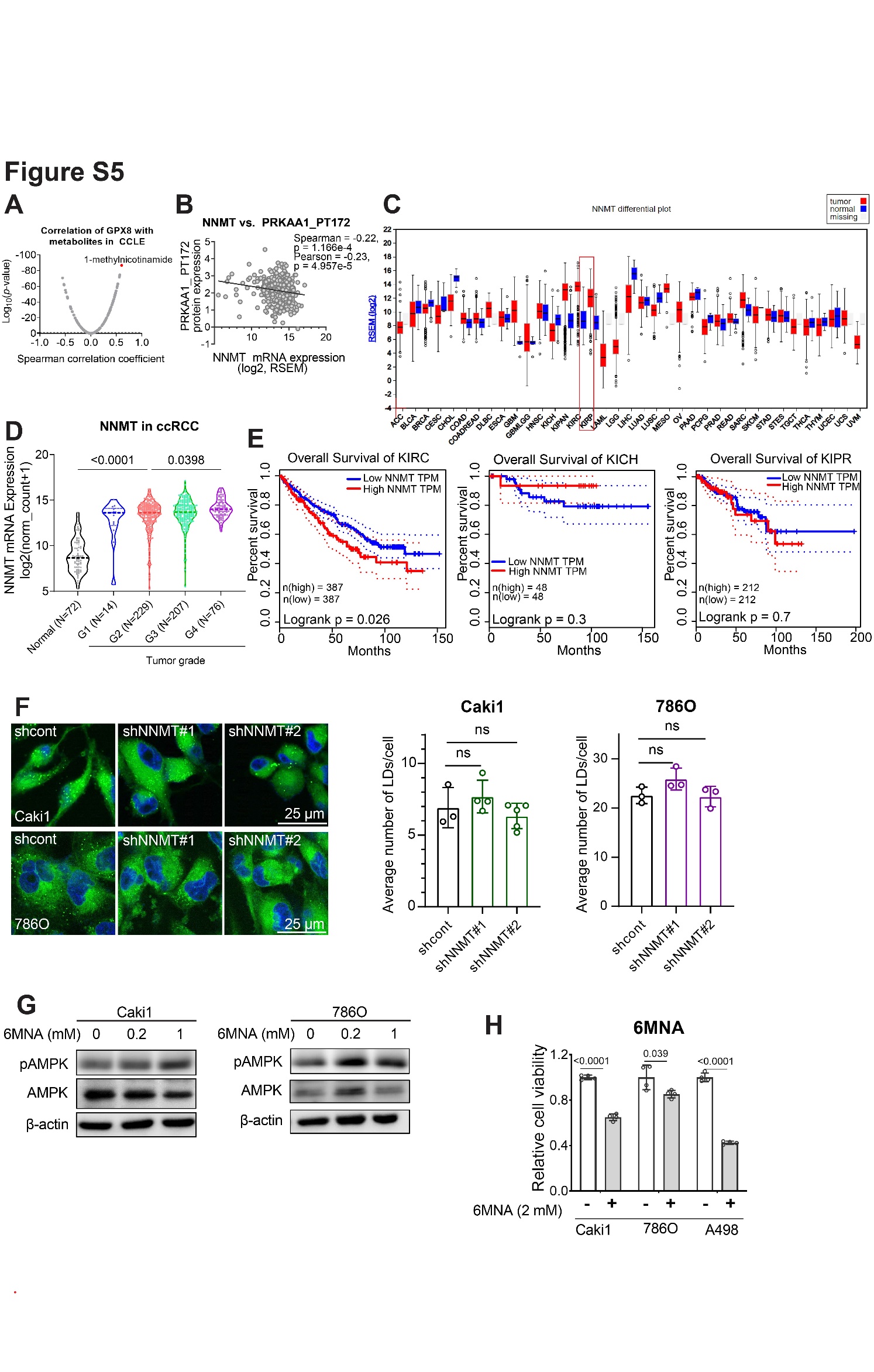


**Figure S5. (Related to Fig. 5) NNMT mediates GPX8’s inhibition of AMPK A,** Correlation between GPX8 gene expression and all the metabolite levels in the CCLE database. Each dot represents one metabolite in the CCLE metabolomics data. The correlation for 1MNA is indicated in red. **B,** Correlation between NNMT mRNA expression and pT172-AMPK protein level in ccRCC patients. **C,** Expression of NNMT across various types of cancer (TCGA-KIRC). The NNMT expression in ccRCC was highlighted in the red box. **D,** NNMT expression according to the tumor grade in ccRCC patients. *P-*values were determined by Mann-Whitney U test. **E,** Overall survival according to the NNMT expression levels in KIRC, KIRP (Kidney renal papillary cell carcinoma), and KICH (Kidney Chromophobe) patients classified with 25% NNMT-high cutoff vs 75% NNMT-low cutoff. The figures were obtained from http://gepia.cancer-pku.cn/. Data from (**B**), (**D**), and (**E**) were obtained from the TCGA dataset. **F**, Representative pictures (left) for the lipid uptake using BODIPY 500/510 C1, C12 staining from shcont vs. shNNMT cell lines (top: Caki1 and bottom: 786O). Quantitation of lipid droplet as in Fig. **3F** (right) (*n* ≥ 3). **G-H**, Effects of 3-day 6MNA treatment: western blot analysis of the total AMPK and phosphorylated AMPK (AMPK α1 (T183) and AMPK α2 (T172)) normalized by β-actin (**G**) and cell viability of ccRCC cell lines (**H**). Data presented in panels (**F**) and (**H**) are means ± SD (*n* ≥ 3). *P*-values were determined by unpaired *t*-test. *ns*, not significant


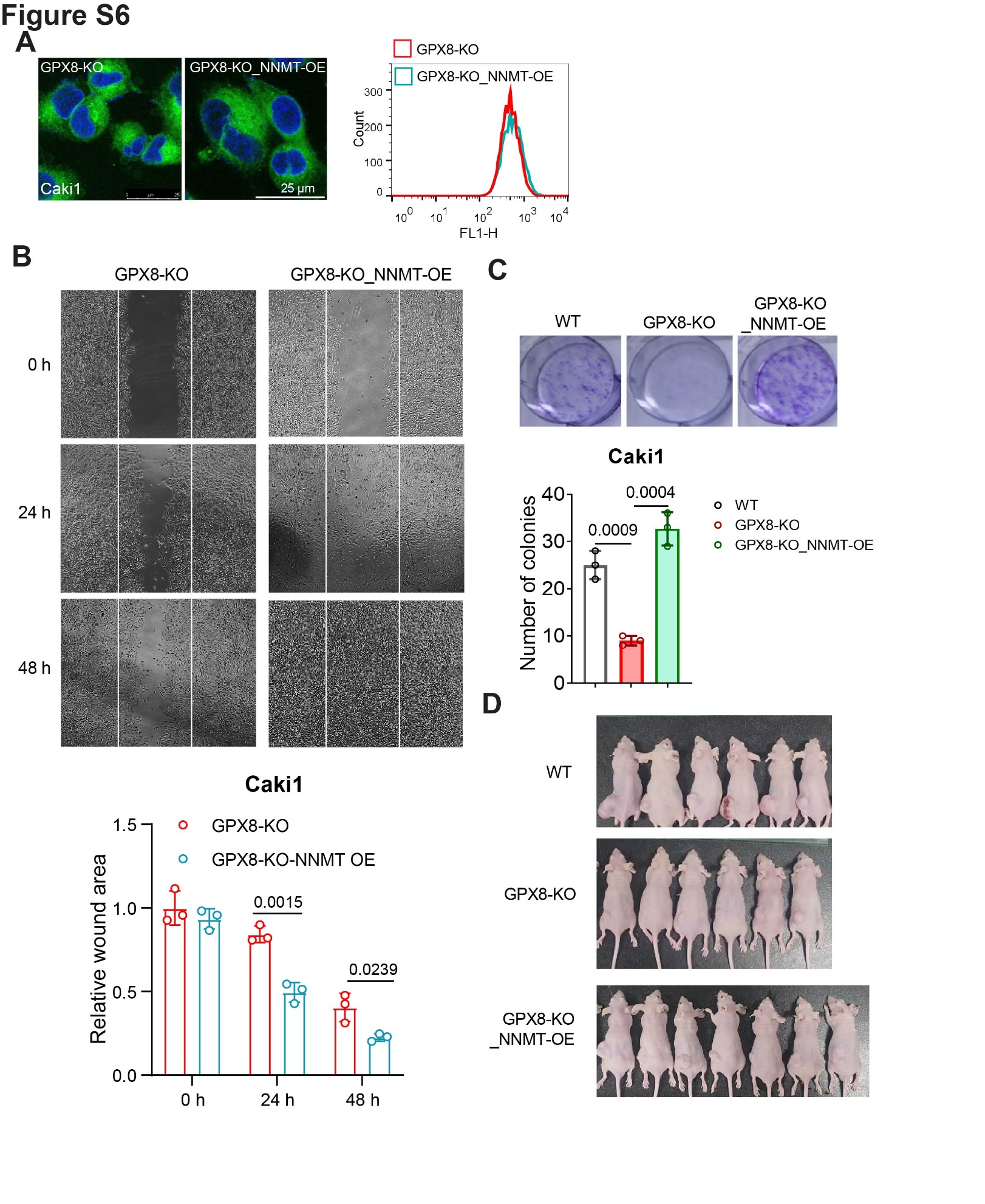


**Figure S6 (Related to Fig. 6). NNMT expression rescues the GPX8 KO phenotype**

**A-C,** Comparison between GPX8-KO Caki1 with and without NNMT OE: Representative pictures for lipid uptake (**A**, left), the fluorescent intensity of lipid uptake measured by flow cytometry (**A**, right), and migrating cells in scratch assay for 0 h, 24 h, and 48 h (top) and quantification of wound areas (bottom) (**B**). Clonogenic assay (**C**) after plating 200 cells in 6-well plates for 48 h (top) and bar graph for the number of colonies (bottom) comparing WT vs. GPX8-KO Caki1 with and without NNMT overexpression. Data presented in panels (**B**) and (**C**) are means ± SD with (*n* = 3). *P*-values were determined by unpaired *t*-test. **D.** Photograph of tumors obtained on the last day of the 5^th^ week after implantation of WT, GPX8-KO, and GPX8-KO with NNMT overexpression (OE) Caki1 cells.


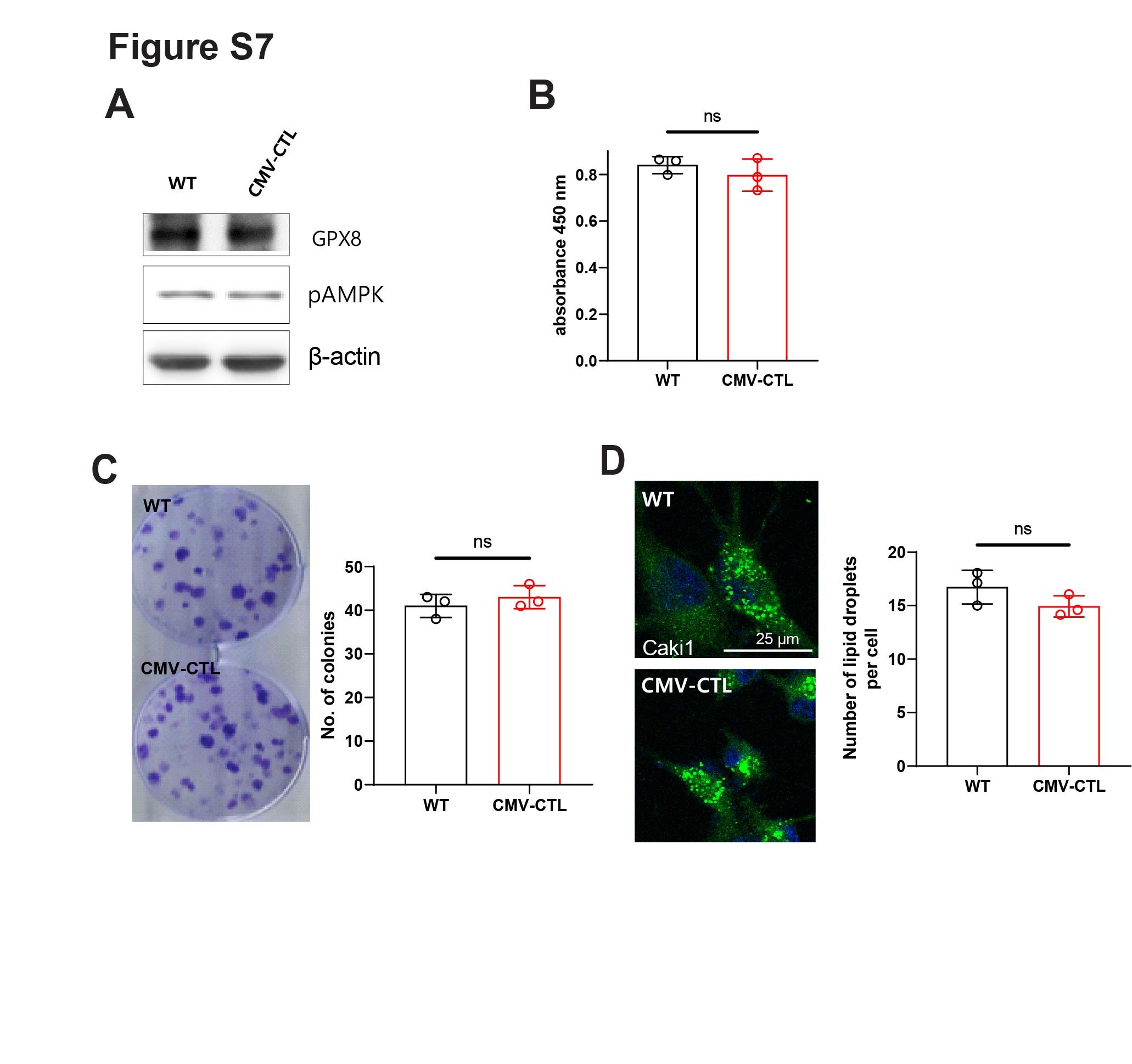


**Figure S7. The effect of empty CMV-CTL vector in Caki1**. Wild type Caki1 and stable Caki1 cells transfected with CMV-CTL (pCMV6-Entry2- Myc-DDK tag) were used in (**A**), (**B**), (**C**) and (**D**). Western blot analysis of GPX8 and phospho-AMPKα1 (T183) and α2 (T172) protein expression (**A**), The cell viability measured by CCK-8 for 2 days (**B**), clonogenic assay after plating 200 cells in 6-well plate for 2 weeks (left) and bar graphs for number of colonies (right) (*n* = 3) (**C**), representative pictures of neutral lipid staining using BODIPY 493/503 (left) and quantitation of lipid droplet as in Fig. **3F** (right) (**D**). Data represented in panels (**B**), (**C**), and (**D**) are means ± SD (*n* = 3). *P*-values were calculated by unpaired *t*-test. *ns*, not significant


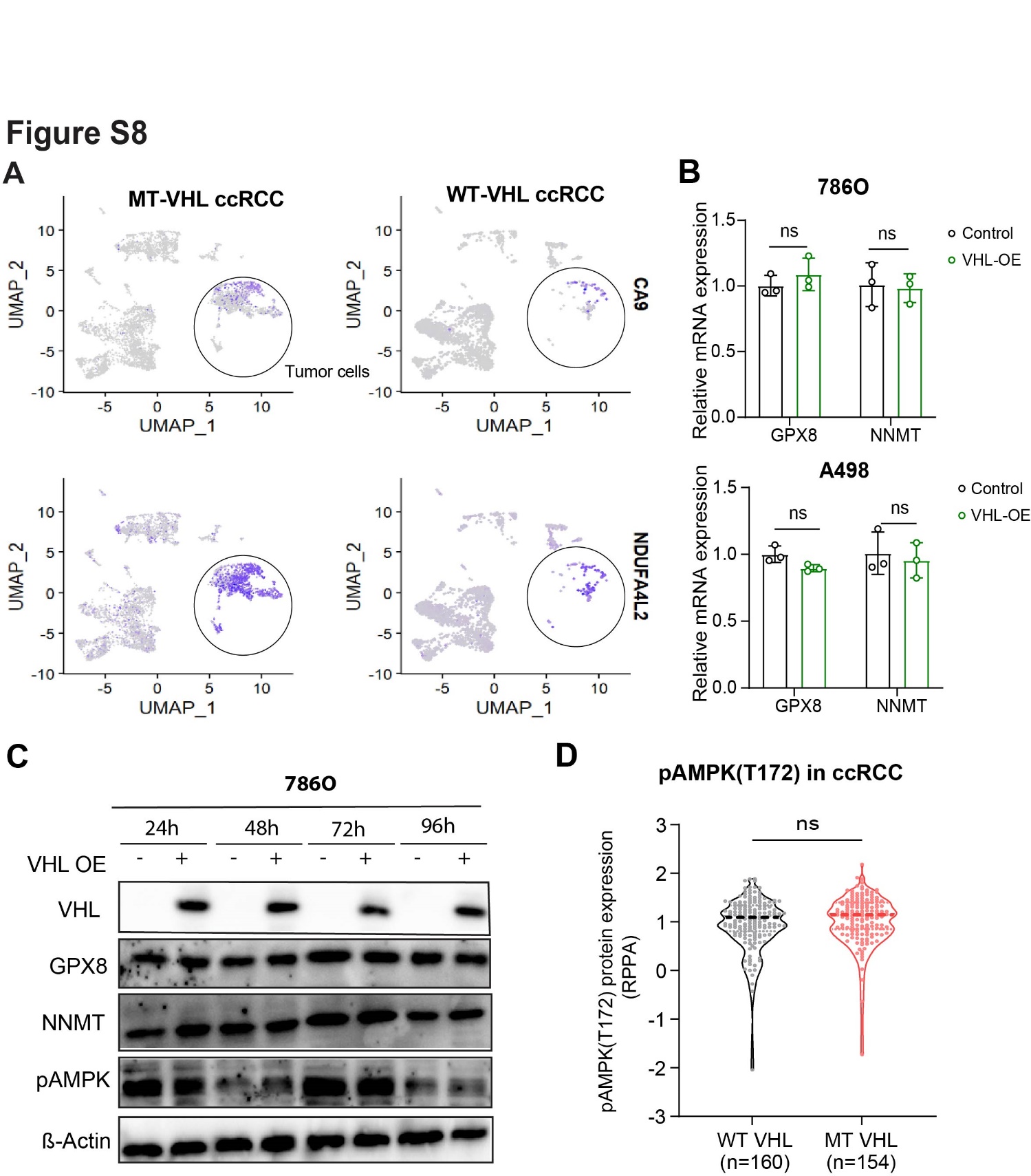


**Figure S8. (Related to Fig. 8) Single-cell RNA-seq data for ccRCC tumors with WT-VHL and MT-VHL**

**A,** Expression of ccRCC markers from sc-RNA seq data used for identifying tumor cells (indicated with black ellipses): CA9 (top) and NDUFA4L2 (bottom). **B**, mRNA expression of GPX8 and NNMT upon stable ectopic expression of VHL in 786O and A498 cells by RT-qPCR. Data presented are means ± SD (*n* = 3). *P*-value was determined by unpaired *t*-test. (**C**) Western blot analysis of VHL, GPX8, NNMT and pAMPKα1 (T183) and α2 (T172) in 786O with VHL stable expression at serial time points after seeding (24 h, 48 h, 72 h, and 96 h). (**D**) Protein expression levels of pAMPK (T172) according to normal and VHL status of tumors in ccRCC patients from the TCGA-KIRC dataset. *ns*, not significant
